# Search for supersymmetry at $\sqrt{s}=13$ TeV in final states with jets and two same-sign leptons or three leptons with the ATLAS detector

**DOI:** 10.1140/epjc/s10052-016-4095-8

**Published:** 2016-05-07

**Authors:** Atlas Collaboration, G. Aad, B. Abbott, J. Abdallah, O. Abdinov, B. Abeloos, R. Aben, M. Abolins, O. S. AbouZeid, H. Abramowicz, H. Abreu, R. Abreu, Y. Abulaiti, B. S. Acharya, L. Adamczyk, D. L. Adams, J. Adelman, S. Adomeit, T. Adye, A. A. Affolder, T. Agatonovic-Jovin, J. Agricola, J. A. Aguilar-Saavedra, S. P. Ahlen, F. Ahmadov, G. Aielli, H. Akerstedt, T. P. A. Åkesson, A. V. Akimov, G. L. Alberghi, J. Albert, S. Albrand, M. J. Alconada Verzini, M. Aleksa, I. N. Aleksandrov, C. Alexa, G. Alexander, T. Alexopoulos, M. Alhroob, G. Alimonti, J. Alison, S. P. Alkire, B. M. M. Allbrooke, B. W. Allen, P. P. Allport, A. Aloisio, A. Alonso, F. Alonso, C. Alpigiani, B. Alvarez Gonzalez, D. Álvarez Piqueras, M. G. Alviggi, B. T. Amadio, K. Amako, Y. Amaral Coutinho, C. Amelung, D. Amidei, S. P. Amor Dos Santos, A. Amorim, S. Amoroso, N. Amram, G. Amundsen, C. Anastopoulos, L. S. Ancu, N. Andari, T. Andeen, C. F. Anders, G. Anders, J. K. Anders, K. J. Anderson, A. Andreazza, V. Andrei, S. Angelidakis, I. Angelozzi, P. Anger, A. Angerami, F. Anghinolfi, A. V. Anisenkov, N. Anjos, A. Annovi, M. Antonelli, A. Antonov, J. Antos, F. Anulli, M. Aoki, L. Aperio Bella, G. Arabidze, Y. Arai, J. P. Araque, A. T. H. Arce, F. A. Arduh, J-F. Arguin, S. Argyropoulos, M. Arik, A. J. Armbruster, L. J. Armitage, O. Arnaez, H. Arnold, M. Arratia, O. Arslan, A. Artamonov, G. Artoni, S. Artz, S. Asai, N. Asbah, A. Ashkenazi, B. Åsman, L. Asquith, K. Assamagan, R. Astalos, M. Atkinson, N. B. Atlay, K. Augsten, G. Avolio, B. Axen, M. K. Ayoub, G. Azuelos, M. A. Baak, A. E. Baas, M. J. Baca, H. Bachacou, K. Bachas, M. Backes, M. Backhaus, P. Bagiacchi, P. Bagnaia, Y. Bai, J. T. Baines, O. K. Baker, E. M. Baldin, P. Balek, T. Balestri, F. Balli, W. K. Balunas, E. Banas, Sw. Banerjee, A. A. E. Bannoura, L. Barak, E. L. Barberio, D. Barberis, M. Barbero, T. Barillari, M. Barisonzi, T. Barklow, N. Barlow, S. L. Barnes, B. M. Barnett, R. M. Barnett, Z. Barnovska, A. Baroncelli, G. Barone, A. J. Barr, L. Barranco Navarro, F. Barreiro, J. Barreiro Guimarães da Costa, R. Bartoldus, A. E. Barton, P. Bartos, A. Basalaev, A. Bassalat, A. Basye, R. L. Bates, S. J. Batista, J. R. Batley, M. Battaglia, M. Bauce, F. Bauer, H. S. Bawa, J. B. Beacham, M. D. Beattie, T. Beau, P. H. Beauchemin, P. Bechtle, H. P. Beck, K. Becker, M. Becker, M. Beckingham, C. Becot, A. J. Beddall, A. Beddall, V. A. Bednyakov, M. Bedognetti, C. P. Bee, L. J. Beemster, T. A. Beermann, M. Begel, J. K. Behr, C. Belanger-Champagne, A. S. Bell, G. Bella, L. Bellagamba, A. Bellerive, M. Bellomo, K. Belotskiy, O. Beltramello, N. L. Belyaev, O. Benary, D. Benchekroun, M. Bender, K. Bendtz, N. Benekos, Y. Benhammou, E. Benhar Noccioli, J. Benitez, J. A. Benitez Garcia, D. P. Benjamin, J. R. Bensinger, S. Bentvelsen, L. Beresford, M. Beretta, D. Berge, E. Bergeaas Kuutmann, N. Berger, F. Berghaus, J. Beringer, S. Berlendis, N. R. Bernard, C. Bernius, F. U. Bernlochner, T. Berry, P. Berta, C. Bertella, G. Bertoli, F. Bertolucci, I. A. Bertram, C. Bertsche, D. Bertsche, G. J. Besjes, O. Bessidskaia Bylund, M. Bessner, N. Besson, C. Betancourt, S. Bethke, A. J. Bevan, W. Bhimji, R. M. Bianchi, L. Bianchini, M. Bianco, O. Biebel, D. Biedermann, R. Bielski, N. V. Biesuz, M. Biglietti, J. Bilbao De Mendizabal, H. Bilokon, M. Bindi, S. Binet, A. Bingul, C. Bini, S. Biondi, D. M. Bjergaard, C. W. Black, J. E. Black, K. M. Black, D. Blackburn, R. E. Blair, J.-B. Blanchard, J. E. Blanco, T. Blazek, I. Bloch, C. Blocker, W. Blum, U. Blumenschein, S. Blunier, G. J. Bobbink, V. S. Bobrovnikov, S. S. Bocchetta, A. Bocci, C. Bock, M. Boehler, D. Boerner, J. A. Bogaerts, D. Bogavac, A. G. Bogdanchikov, C. Bohm, V. Boisvert, T. Bold, V. Boldea, A. S. Boldyrev, M. Bomben, M. Bona, M. Boonekamp, A. Borisov, G. Borissov, J. Bortfeldt, D. Bortoletto, V. Bortolotto, K. Bos, D. Boscherini, M. Bosman, J. D. Bossio Sola, J. Boudreau, J. Bouffard, E. V. Bouhova-Thacker, D. Boumediene, C. Bourdarios, N. Bousson, S. K. Boutle, A. Boveia, J. Boyd, I. R. Boyko, J. Bracinik, A. Brandt, G. Brandt, O. Brandt, U. Bratzler, B. Brau, J. E. Brau, H. M. Braun, W. D. Breaden Madden, K. Brendlinger, A. J. Brennan, L. Brenner, R. Brenner, S. Bressler, T. M. Bristow, D. Britton, D. Britzger, F. M. Brochu, I. Brock, R. Brock, G. Brooijmans, T. Brooks, W. K. Brooks, J. Brosamer, E. Brost, J.H Broughton, P. A. Bruckman de Renstrom, D. Bruncko, R. Bruneliere, A. Bruni, G. Bruni, BH Brunt, M. Bruschi, N. Bruscino, P. Bryant, L. Bryngemark, T. Buanes, Q. Buat, P. Buchholz, A. G. Buckley, I. A. Budagov, F. Buehrer, M. K. Bugge, O. Bulekov, D. Bullock, H. Burckhart, S. Burdin, C. D. Burgard, B. Burghgrave, K. Burka, S. Burke, I. Burmeister, E. Busato, D. Büscher, V. Büscher, P. Bussey, J. M. Butler, A. I. Butt, C. M. Buttar, J. M. Butterworth, P. Butti, W. Buttinger, A. Buzatu, A. R. Buzykaev, S. Cabrera Urbán, D. Caforio, V. M. Cairo, O. Cakir, N. Calace, P. Calafiura, A. Calandri, G. Calderini, P. Calfayan, L. P. Caloba, D. Calvet, S. Calvet, T. P. Calvet, R. Camacho Toro, S. Camarda, P. Camarri, D. Cameron, R. Caminal Armadans, C. Camincher, S. Campana, M. Campanelli, A. Campoverde, V. Canale, A. Canepa, M. Cano Bret, J. Cantero, R. Cantrill, T. Cao, M. D. M. Capeans Garrido, I. Caprini, M. Caprini, M. Capua, R. Caputo, R. M. Carbone, R. Cardarelli, F. Cardillo, T. Carli, G. Carlino, L. Carminati, S. Caron, E. Carquin, G. D. Carrillo-Montoya, J. R. Carter, J. Carvalho, D. Casadei, M. P. Casado, M. Casolino, D. W. Casper, E. Castaneda-Miranda, A. Castelli, V. Castillo Gimenez, N. F. Castro, A. Catinaccio, J. R. Catmore, A. Cattai, J. Caudron, V. Cavaliere, D. Cavalli, M. Cavalli-Sforza, V. Cavasinni, F. Ceradini, L. Cerda Alberich, B. C. Cerio, A. S. Cerqueira, A. Cerri, L. Cerrito, F. Cerutti, M. Cerv, A. Cervelli, S. A. Cetin, A. Chafaq, D. Chakraborty, I. Chalupkova, S. K. Chan, Y. L. Chan, P. Chang, J. D. Chapman, D. G. Charlton, A. Chatterjee, C. C. Chau, C. A. Chavez Barajas, S. Che, S. Cheatham, A. Chegwidden, S. Chekanov, S. V. Chekulaev, G. A. Chelkov, M. A. Chelstowska, C. Chen, H. Chen, K. Chen, S. Chen, S. Chen, X. Chen, Y. Chen, H. C. Cheng, H.J Cheng, Y. Cheng, A. Cheplakov, E. Cheremushkina, R. Cherkaoui El Moursli, V. Chernyatin, E. Cheu, L. Chevalier, V. Chiarella, G. Chiarelli, G. Chiodini, A. S. Chisholm, A. Chitan, M. V. Chizhov, K. Choi, A. R. Chomont, S. Chouridou, B. K. B. Chow, V. Christodoulou, D. Chromek-Burckhart, J. Chudoba, A. J. Chuinard, J. J. Chwastowski, L. Chytka, G. Ciapetti, A. K. Ciftci, D. Cinca, V. Cindro, I. A. Cioara, A. Ciocio, F. Cirotto, Z. H. Citron, M. Ciubancan, A. Clark, B. L. Clark, P. J. Clark, R. N. Clarke, C. Clement, Y. Coadou, M. Cobal, A. Coccaro, J. Cochran, L. Coffey, L. Colasurdo, B. Cole, S. Cole, A. P. Colijn, J. Collot, T. Colombo, G. Compostella, P. Conde Muiño, E. Coniavitis, S. H. Connell, I. A. Connelly, V. Consorti, S. Constantinescu, C. Conta, G. Conti, F. Conventi, M. Cooke, B. D. Cooper, A. M. Cooper-Sarkar, T. Cornelissen, M. Corradi, F. Corriveau, A. Corso-Radu, A. Cortes-Gonzalez, G. Cortiana, G. Costa, M. J. Costa, D. Costanzo, G. Cottin, G. Cowan, B. E. Cox, K. Cranmer, S. J. Crawley, G. Cree, S. Crépé-Renaudin, F. Crescioli, W. A. Cribbs, M. Crispin Ortuzar, M. Cristinziani, V. Croft, G. Crosetti, T. Cuhadar Donszelmann, J. Cummings, M. Curatolo, J. Cúth, C. Cuthbert, H. Czirr, P. Czodrowski, S. D’Auria, M. D’Onofrio, M. J. Da Cunha Sargedas De Sousa, C. Da Via, W. Dabrowski, T. Dai, O. Dale, F. Dallaire, C. Dallapiccola, M. Dam, J. R. Dandoy, N. P. Dang, A. C. Daniells, N. S. Dann, M. Danninger, M. Dano Hoffmann, V. Dao, G. Darbo, S. Darmora, J. Dassoulas, A. Dattagupta, W. Davey, C. David, T. Davidek, M. Davies, P. Davison, Y. Davygora, E. Dawe, I. Dawson, R. K. Daya-Ishmukhametova, K. De, R. de Asmundis, A. De Benedetti, S. De Castro, S. De Cecco, N. De Groot, P. de Jong, H. De la Torre, F. De Lorenzi, D. De Pedis, A. De Salvo, U. De Sanctis, A. De Santo, J. B. De Vivie De Regie, W. J. Dearnaley, R. Debbe, C. Debenedetti, D. V. Dedovich, I. Deigaard, J. Del Peso, T. Del Prete, D. Delgove, F. Deliot, C. M. Delitzsch, M. Deliyergiyev, A. Dell’Acqua, L. Dell’Asta, M. Dell’Orso, M. Della Pietra, D. della Volpe, M. Delmastro, P. A. Delsart, C. Deluca, D. A. DeMarco, S. Demers, M. Demichev, A. Demilly, S. P. Denisov, D. Denysiuk, D. Derendarz, J. E. Derkaoui, F. Derue, P. Dervan, K. Desch, C. Deterre, K. Dette, P. O. Deviveiros, A. Dewhurst, S. Dhaliwal, A. Di Ciaccio, L. Di Ciaccio, W. K. Di Clemente, C. Di Donato, A. Di Girolamo, B. Di Girolamo, B. Di Micco, R. Di Nardo, A. Di Simone, R. Di Sipio, D. Di Valentino, C. Diaconu, M. Diamond, F. A. Dias, M. A. Diaz, E. B. Diehl, J. Dietrich, S. Diglio, A. Dimitrievska, J. Dingfelder, P. Dita, S. Dita, F. Dittus, F. Djama, T. Djobava, J. I. Djuvsland, M. A. B. do Vale, D. Dobos, M. Dobre, C. Doglioni, T. Dohmae, J. Dolejsi, Z. Dolezal, B. A. Dolgoshein, M. Donadelli, S. Donati, P. Dondero, J. Donini, J. Dopke, A. Doria, M. T. Dova, A. T. Doyle, E. Drechsler, M. Dris, Y. Du, J. Duarte-Campderros, E. Duchovni, G. Duckeck, O. A. Ducu, D. Duda, A. Dudarev, L. Duflot, L. Duguid, M. Dührssen, M. Dunford, H. Duran Yildiz, M. Düren, A. Durglishvili, D. Duschinger, B. Dutta, M. Dyndal, C. Eckardt, K. M. Ecker, R. C. Edgar, W. Edson, N. C. Edwards, T. Eifert, G. Eigen, K. Einsweiler, T. Ekelof, M. El Kacimi, V. Ellajosyula, M. Ellert, S. Elles, F. Ellinghaus, A. A. Elliot, N. Ellis, J. Elmsheuser, M. Elsing, D. Emeliyanov, Y. Enari, O. C. Endner, M. Endo, J. S. Ennis, J. Erdmann, A. Ereditato, G. Ernis, J. Ernst, M. Ernst, S. Errede, E. Ertel, M. Escalier, H. Esch, C. Escobar, B. Esposito, A. I. Etienvre, E. Etzion, H. Evans, A. Ezhilov, F. Fabbri, L. Fabbri, G. Facini, R. M. Fakhrutdinov, S. Falciano, R. J. Falla, J. Faltova, Y. Fang, M. Fanti, A. Farbin, A. Farilla, C. Farina, T. Farooque, S. Farrell, S. M. Farrington, P. Farthouat, F. Fassi, P. Fassnacht, D. Fassouliotis, M. Faucci Giannelli, A. Favareto, W. J. Fawcett, L. Fayard, O. L. Fedin, W. Fedorko, S. Feigl, L. Feligioni, C. Feng, E. J. Feng, H. Feng, A. B. Fenyuk, L. Feremenga, P. Fernandez Martinez, S. Fernandez Perez, J. Ferrando, A. Ferrari, P. Ferrari, R. Ferrari, D. E. Ferreira de Lima, A. Ferrer, D. Ferrere, C. Ferretti, A. Ferretto Parodi, F. Fiedler, A. Filipčič, M. Filipuzzi, F. Filthaut, M. Fincke-Keeler, K. D. Finelli, M. C. N. Fiolhais, L. Fiorini, A. Firan, A. Fischer, C. Fischer, J. Fischer, W. C. Fisher, N. Flaschel, I. Fleck, P. Fleischmann, G. T. Fletcher, G. Fletcher, R. R. M. Fletcher, T. Flick, A. Floderus, L. R. Flores Castillo, M. J. Flowerdew, G. T. Forcolin, A. Formica, A. Forti, A. G. Foster, D. Fournier, H. Fox, S. Fracchia, P. Francavilla, M. Franchini, D. Francis, L. Franconi, M. Franklin, M. Frate, M. Fraternali, D. Freeborn, S. M. Fressard-Batraneanu, F. Friedrich, D. Froidevaux, J. A. Frost, C. Fukunaga, E. Fullana Torregrosa, T. Fusayasu, J. Fuster, C. Gabaldon, O. Gabizon, A. Gabrielli, A. Gabrielli, G. P. Gach, S. Gadatsch, S. Gadomski, G. Gagliardi, L. G. Gagnon, P. Gagnon, C. Galea, B. Galhardo, E. J. Gallas, B. J. Gallop, P. Gallus, G. Galster, K. K. Gan, J. Gao, Y. Gao, Y. S. Gao, F. M. Garay Walls, C. García, J. E. García Navarro, M. Garcia-Sciveres, R. W. Gardner, N. Garelli, V. Garonne, A. Gascon Bravo, C. Gatti, A. Gaudiello, G. Gaudio, B. Gaur, L. Gauthier, I. L. Gavrilenko, C. Gay, G. Gaycken, E. N. Gazis, Z. Gecse, C. N. P. Gee, Ch. Geich-Gimbel, M. P. Geisler, C. Gemme, M. H. Genest, C. Geng, S. Gentile, S. George, D. Gerbaudo, A. Gershon, S. Ghasemi, H. Ghazlane, B. Giacobbe, S. Giagu, P. Giannetti, B. Gibbard, S. M. Gibson, M. Gignac, M. Gilchriese, T. P. S. Gillam, D. Gillberg, G. Gilles, D. M. Gingrich, N. Giokaris, M. P. Giordani, F. M. Giorgi, F. M. Giorgi, P. F. Giraud, P. Giromini, D. Giugni, C. Giuliani, M. Giulini, B. K. Gjelsten, S. Gkaitatzis, I. Gkialas, E. L. Gkougkousis, L. K. Gladilin, C. Glasman, J. Glatzer, P. C. F. Glaysher, A. Glazov, M. Goblirsch-Kolb, J. Godlewski, S. Goldfarb, T. Golling, D. Golubkov, A. Gomes, R. Gonçalo, J. Goncalves Pinto Firmino Da Costa, L. Gonella, A. Gongadze, S. González de la Hoz, G. Gonzalez Parra, S. Gonzalez-Sevilla, L. Goossens, P. A. Gorbounov, H. A. Gordon, I. Gorelov, B. Gorini, E. Gorini, A. Gorišek, E. Gornicki, A. T. Goshaw, C. Gössling, M. I. Gostkin, C. R. Goudet, D. Goujdami, A. G. Goussiou, N. Govender, E. Gozani, L. Graber, I. Grabowska-Bold, P. O. J. Gradin, P. Grafström, J. Gramling, E. Gramstad, S. Grancagnolo, V. Gratchev, H. M. Gray, E. Graziani, Z. D. Greenwood, C. Grefe, K. Gregersen, I. M. Gregor, P. Grenier, K. Grevtsov, J. Griffiths, A. A. Grillo, K. Grimm, S. Grinstein, Ph. Gris, J.-F. Grivaz, S. Groh, J. P. Grohs, E. Gross, J. Grosse-Knetter, G. C. Grossi, Z. J. Grout, L. Guan, W. Guan, J. Guenther, F. Guescini, D. Guest, O. Gueta, E. Guido, T. Guillemin, S. Guindon, U. Gul, C. Gumpert, J. Guo, Y. Guo, S. Gupta, G. Gustavino, P. Gutierrez, N. G. Gutierrez Ortiz, C. Gutschow, C. Guyot, C. Gwenlan, C. B. Gwilliam, A. Haas, C. Haber, H. K. Hadavand, N. Haddad, A. Hadef, P. Haefner, S. Hageböck, Z. Hajduk, H. Hakobyan, M. Haleem, J. Haley, D. Hall, G. Halladjian, G. D. Hallewell, K. Hamacher, P. Hamal, K. Hamano, A. Hamilton, G. N. Hamity, P. G. Hamnett, L. Han, K. Hanagaki, K. Hanawa, M. Hance, B. Haney, P. Hanke, R. Hanna, J. B. Hansen, J. D. Hansen, M. C. Hansen, P. H. Hansen, K. Hara, A. S. Hard, T. Harenberg, F. Hariri, S. Harkusha, R. D. Harrington, P. F. Harrison, F. Hartjes, M. Hasegawa, Y. Hasegawa, A. Hasib, S. Hassani, S. Haug, R. Hauser, L. Hauswald, M. Havranek, C. M. Hawkes, R. J. Hawkings, A. D. Hawkins, D. Hayden, C. P. Hays, J. M. Hays, H. S. Hayward, S. J. Haywood, S. J. Head, T. Heck, V. Hedberg, L. Heelan, S. Heim, T. Heim, B. Heinemann, J. J. Heinrich, L. Heinrich, C. Heinz, J. Hejbal, L. Helary, S. Hellman, C. Helsens, J. Henderson, R. C. W. Henderson, Y. Heng, S. Henkelmann, A. M. Henriques Correia, S. Henrot-Versille, G. H. Herbert, Y. Hernández Jiménez, G. Herten, R. Hertenberger, L. Hervas, G. G. Hesketh, N. P. Hessey, J. W. Hetherly, R. Hickling, E. Higón-Rodriguez, E. Hill, J. C. Hill, K. H. Hiller, S. J. Hillier, I. Hinchliffe, E. Hines, R. R. Hinman, M. Hirose, D. Hirschbuehl, J. Hobbs, N. Hod, M. C. Hodgkinson, P. Hodgson, A. Hoecker, M. R. Hoeferkamp, F. Hoenig, M. Hohlfeld, D. Hohn, T. R. Holmes, M. Homann, T. M. Hong, B. H. Hooberman, W. H. Hopkins, Y. Horii, A. J. Horton, J-Y. Hostachy, S. Hou, A. Hoummada, J. Howard, J. Howarth, M. Hrabovsky, I. Hristova, J. Hrivnac, T. Hryn’ova, A. Hrynevich, C. Hsu, P. J. Hsu, S.-C. Hsu, D. Hu, Q. Hu, Y. Huang, Z. Hubacek, F. Hubaut, F. Huegging, T. B. Huffman, E. W. Hughes, G. Hughes, M. Huhtinen, T. A. Hülsing, N. Huseynov, J. Huston, J. Huth, G. Iacobucci, G. Iakovidis, I. Ibragimov, L. Iconomidou-Fayard, E. Ideal, Z. Idrissi, P. Iengo, O. Igonkina, T. Iizawa, Y. Ikegami, M. Ikeno, Y. Ilchenko, D. Iliadis, N. Ilic, T. Ince, G. Introzzi, P. Ioannou, M. Iodice, K. Iordanidou, V. Ippolito, A. Irles Quiles, C. Isaksson, M. Ishino, M. Ishitsuka, R. Ishmukhametov, C. Issever, S. Istin, F. Ito, J. M. Iturbe Ponce, R. Iuppa, J. Ivarsson, W. Iwanski, H. Iwasaki, J. M. Izen, V. Izzo, S. Jabbar, B. Jackson, M. Jackson, P. Jackson, V. Jain, K. B. Jakobi, K. Jakobs, S. Jakobsen, T. Jakoubek, D. O. Jamin, D. K. Jana, E. Jansen, R. Jansky, J. Janssen, M. Janus, G. Jarlskog, N. Javadov, T. Javůrek, F. Jeanneau, L. Jeanty, J. Jejelava, G.-Y. Jeng, D. Jennens, P. Jenni, J. Jentzsch, C. Jeske, S. Jézéquel, H. Ji, J. Jia, H. Jiang, Y. Jiang, S. Jiggins, J. Jimenez Pena, S. Jin, A. Jinaru, O. Jinnouchi, P. Johansson, K. A. Johns, W. J. Johnson, K. Jon-And, G. Jones, R. W. L. Jones, S. Jones, T. J. Jones, J. Jongmanns, P. M. Jorge, J. Jovicevic, X. Ju, A. Juste Rozas, M. K. Köhler, A. Kaczmarska, M. Kado, H. Kagan, M. Kagan, S. J. Kahn, E. Kajomovitz, C. W. Kalderon, A. Kaluza, S. Kama, A. Kamenshchikov, N. Kanaya, S. Kaneti, V. A. Kantserov, J. Kanzaki, B. Kaplan, L. S. Kaplan, A. Kapliy, D. Kar, K. Karakostas, A. Karamaoun, N. Karastathis, M. J. Kareem, E. Karentzos, M. Karnevskiy, S. N. Karpov, Z. M. Karpova, K. Karthik, V. Kartvelishvili, A. N. Karyukhin, K. Kasahara, L. Kashif, R. D. Kass, A. Kastanas, Y. Kataoka, C. Kato, A. Katre, J. Katzy, K. Kawagoe, T. Kawamoto, G. Kawamura, S. Kazama, V. F. Kazanin, R. Keeler, R. Kehoe, J. S. Keller, J. J. Kempster, K Kentaro, H. Keoshkerian, O. Kepka, B. P. Kerševan, S. Kersten, R. A. Keyes, F. Khalil-zada, H. Khandanyan, A. Khanov, A. G. Kharlamov, T. J. Khoo, V. Khovanskiy, E. Khramov, J. Khubua, S. Kido, H. Y. Kim, S. H. Kim, Y. K. Kim, N. Kimura, O. M. Kind, B. T. King, M. King, S. B. King, J. Kirk, A. E. Kiryunin, T. Kishimoto, D. Kisielewska, F. Kiss, K. Kiuchi, O. Kivernyk, E. Kladiva, M. H. Klein, M. Klein, U. Klein, K. Kleinknecht, P. Klimek, A. Klimentov, R. Klingenberg, J. A. Klinger, T. Klioutchnikova, E.-E. Kluge, P. Kluit, S. Kluth, J. Knapik, E. Kneringer, E. B. F. G. Knoops, A. Knue, A. Kobayashi, D. Kobayashi, T. Kobayashi, M. Kobel, M. Kocian, P. Kodys, T. Koffas, E. Koffeman, L. A. Kogan, T. Kohriki, T. Koi, H. Kolanoski, M. Kolb, I. Koletsou, A. A. Komar, Y. Komori, T. Kondo, N. Kondrashova, K. Köneke, A. C. König, T. Kono, R. Konoplich, N. Konstantinidis, R. Kopeliansky, S. Koperny, L. Köpke, A. K. Kopp, K. Korcyl, K. Kordas, A. Korn, A. A. Korol, I. Korolkov, E. V. Korolkova, O. Kortner, S. Kortner, T. Kosek, V. V. Kostyukhin, V. M. Kotov, A. Kotwal, A. Kourkoumeli-Charalampidi, C. Kourkoumelis, V. Kouskoura, A. Koutsman, A. B. Kowalewska, R. Kowalewski, T. Z. Kowalski, W. Kozanecki, A. S. Kozhin, V. A. Kramarenko, G. Kramberger, D. Krasnopevtsev, M. W. Krasny, A. Krasznahorkay, J. K. Kraus, A. Kravchenko, M. Kretz, J. Kretzschmar, K. Kreutzfeldt, P. Krieger, K. Krizka, K. Kroeninger, H. Kroha, J. Kroll, J. Kroseberg, J. Krstic, U. Kruchonak, H. Krüger, N. Krumnack, A. Kruse, M. C. Kruse, M. Kruskal, T. Kubota, H. Kucuk, S. Kuday, J. T. Kuechler, S. Kuehn, A. Kugel, F. Kuger, A. Kuhl, T. Kuhl, V. Kukhtin, R. Kukla, Y. Kulchitsky, S. Kuleshov, M. Kuna, T. Kunigo, A. Kupco, H. Kurashige, Y. A. Kurochkin, V. Kus, E. S. Kuwertz, M. Kuze, J. Kvita, T. Kwan, D. Kyriazopoulos, A. La Rosa, J. L. La Rosa Navarro, L. La Rotonda, C. Lacasta, F. Lacava, J. Lacey, H. Lacker, D. Lacour, V. R. Lacuesta, E. Ladygin, R. Lafaye, B. Laforge, T. Lagouri, S. Lai, S. Lammers, W. Lampl, E. Lançon, U. Landgraf, M. P. J. Landon, V. S. Lang, J. C. Lange, A. J. Lankford, F. Lanni, K. Lantzsch, A. Lanza, S. Laplace, C. Lapoire, J. F. Laporte, T. Lari, F. Lasagni Manghi, M. Lassnig, P. Laurelli, W. Lavrijsen, A. T. Law, P. Laycock, T. Lazovich, M. Lazzaroni, O. Le Dortz, E. Le Guirriec, E. Le Menedeu, E. P. Le Quilleuc, M. LeBlanc, T. LeCompte, F. Ledroit-Guillon, C. A. Lee, S. C. Lee, L. Lee, G. Lefebvre, M. Lefebvre, F. Legger, C. Leggett, A. Lehan, G. Lehmann Miotto, X. Lei, W. A. Leight, A. Leisos, A. G. Leister, M. A. L. Leite, R. Leitner, D. Lellouch, B. Lemmer, K. J. C. Leney, T. Lenz, B. Lenzi, R. Leone, S. Leone, C. Leonidopoulos, S. Leontsinis, G. Lerner, C. Leroy, A. A. J. Lesage, C. G. Lester, M. Levchenko, J. Levêque, D. Levin, L. J. Levinson, M. Levy, A. M. Leyko, M. Leyton, B. Li, H. Li, H. L. Li, L. Li, L. Li, Q. Li, S. Li, X. Li, Y. Li, Z. Liang, H. Liao, B. Liberti, A. Liblong, P. Lichard, K. Lie, J. Liebal, W. Liebig, C. Limbach, A. Limosani, S. C. Lin, T. H. Lin, B. E. Lindquist, E. Lipeles, A. Lipniacka, M. Lisovyi, T. M. Liss, D. Lissauer, A. Lister, A. M. Litke, B. Liu, D. Liu, H. Liu, H. Liu, J. Liu, J. B. Liu, K. Liu, L. Liu, M. Liu, M. Liu, Y. L. Liu, Y. Liu, M. Livan, A. Lleres, J. Llorente Merino, S. L. Lloyd, F. Lo Sterzo, E. Lobodzinska, P. Loch, W. S. Lockman, F. K. Loebinger, A. E. Loevschall-Jensen, K. M. Loew, A. Loginov, T. Lohse, K. Lohwasser, M. Lokajicek, B. A. Long, J. D. Long, R. E. Long, L. Longo, K. A. Looper, L. Lopes, D. Lopez Mateos, B. Lopez Paredes, I. Lopez Paz, A. Lopez Solis, J. Lorenz, N. Lorenzo Martinez, M. Losada, P. J. Lösel, X. Lou, A. Lounis, J. Love, P. A. Love, H. Lu, N. Lu, H. J. Lubatti, C. Luci, A. Lucotte, C. Luedtke, F. Luehring, W. Lukas, L. Luminari, O. Lundberg, B. Lund-Jensen, D. Lynn, R. Lysak, E. Lytken, V. Lyubushkin, H. Ma, L. L. Ma, G. Maccarrone, A. Macchiolo, C. M. Macdonald, B. Maček, J. Machado Miguens, D. Madaffari, R. Madar, H. J. Maddocks, W. F. Mader, A. Madsen, J. Maeda, S. Maeland, T. Maeno, A. Maevskiy, E. Magradze, J. Mahlstedt, C. Maiani, C. Maidantchik, A. A. Maier, T. Maier, A. Maio, S. Majewski, Y. Makida, N. Makovec, B. Malaescu, Pa. Malecki, V. P. Maleev, F. Malek, U. Mallik, D. Malon, C. Malone, S. Maltezos, V. M. Malyshev, S. Malyukov, J. Mamuzic, G. Mancini, B. Mandelli, L. Mandelli, I. Mandić, J. Maneira, L. Manhaes de Andrade Filho, J. Manjarres Ramos, A. Mann, B. Mansoulie, R. Mantifel, M. Mantoani, S. Manzoni, L. Mapelli, G. Marceca, L. March, G. Marchiori, M. Marcisovsky, M. Marjanovic, D. E. Marley, F. Marroquim, S. P. Marsden, Z. Marshall, L. F. Marti, S. Marti-Garcia, B. Martin, T. A. Martin, V. J. Martin, B. Martin dit Latour, M. Martinez, S. Martin-Haugh, V. S. Martoiu, A. C. Martyniuk, M. Marx, F. Marzano, A. Marzin, L. Masetti, T. Mashimo, R. Mashinistov, J. Masik, A. L. Maslennikov, I. Massa, L. Massa, P. Mastrandrea, A. Mastroberardino, T. Masubuchi, P. Mättig, J. Mattmann, J. Maurer, S. J. Maxfield, D. A. Maximov, R. Mazini, S. M. Mazza, N. C. Mc Fadden, G. Mc Goldrick, S. P. Mc Kee, A. McCarn, R. L. McCarthy, T. G. McCarthy, L. I. McClymont, K. W. McFarlane, J. A. Mcfayden, G. Mchedlidze, S. J. McMahon, R. A. McPherson, M. Medinnis, S. Meehan, S. Mehlhase, A. Mehta, K. Meier, C. Meineck, B. Meirose, B. R. Mellado Garcia, F. Meloni, A. Mengarelli, S. Menke, E. Meoni, K. M. Mercurio, S. Mergelmeyer, P. Mermod, L. Merola, C. Meroni, F. S. Merritt, A. Messina, J. Metcalfe, A. S. Mete, C. Meyer, C. Meyer, J-P. Meyer, J. Meyer, H. Meyer Zu Theenhausen, R. P. Middleton, S. Miglioranzi, L. Mijović, G. Mikenberg, M. Mikestikova, M. Mikuž, M. Milesi, A. Milic, D. W. Miller, C. Mills, A. Milov, D. A. Milstead, A. A. Minaenko, Y. Minami, I. A. Minashvili, A. I. Mincer, B. Mindur, M. Mineev, Y. Ming, L. M. Mir, K. P. Mistry, T. Mitani, J. Mitrevski, V. A. Mitsou, A. Miucci, P. S. Miyagawa, J. U. Mjörnmark, T. Moa, K. Mochizuki, S. Mohapatra, W. Mohr, S. Molander, R. Moles-Valls, R. Monden, M. C. Mondragon, K. Mönig, J. Monk, E. Monnier, A. Montalbano, J. Montejo Berlingen, F. Monticelli, S. Monzani, R. W. Moore, N. Morange, D. Moreno, M. Moreno Llácer, P. Morettini, D. Mori, T. Mori, M. Morii, M. Morinaga, V. Morisbak, S. Moritz, A. K. Morley, G. Mornacchi, J. D. Morris, S. S. Mortensen, L. Morvaj, M. Mosidze, J. Moss, K. Motohashi, R. Mount, E. Mountricha, S. V. Mouraviev, E. J. W. Moyse, S. Muanza, R. D. Mudd, F. Mueller, J. Mueller, R. S. P. Mueller, T. Mueller, D. Muenstermann, P. Mullen, G. A. Mullier, F. J. Munoz Sanchez, J. A. Murillo Quijada, W. J. Murray, H. Musheghyan, A. G. Myagkov, M. Myska, B. P. Nachman, O. Nackenhorst, J. Nadal, K. Nagai, R. Nagai, Y. Nagai, K. Nagano, Y. Nagasaka, K. Nagata, M. Nagel, E. Nagy, A. M. Nairz, Y. Nakahama, K. Nakamura, T. Nakamura, I. Nakano, H. Namasivayam, R. F. Naranjo Garcia, R. Narayan, D. I. Narrias Villar, I. Naryshkin, T. Naumann, G. Navarro, R. Nayyar, H. A. Neal, P. Yu. Nechaeva, T. J. Neep, P. D. Nef, A. Negri, M. Negrini, S. Nektarijevic, C. Nellist, A. Nelson, S. Nemecek, P. Nemethy, A. A. Nepomuceno, M. Nessi, M. S. Neubauer, M. Neumann, R. M. Neves, P. Nevski, P. R. Newman, D. H. Nguyen, R. B. Nickerson, R. Nicolaidou, B. Nicquevert, J. Nielsen, A. Nikiforov, V. Nikolaenko, I. Nikolic-Audit, K. Nikolopoulos, J. K. Nilsen, P. Nilsson, Y. Ninomiya, A. Nisati, R. Nisius, T. Nobe, L. Nodulman, M. Nomachi, I. Nomidis, T. Nooney, S. Norberg, M. Nordberg, N. Norjoharuddeen, O. Novgorodova, S. Nowak, M. Nozaki, L. Nozka, K. Ntekas, E. Nurse, F. Nuti, F. O’grady, D. C. O’Neil, A. A. O’Rourke, V. O’Shea, F. G. Oakham, H. Oberlack, T. Obermann, J. Ocariz, A. Ochi, I. Ochoa, J. P. Ochoa-Ricoux, S. Oda, S. Odaka, H. Ogren, A. Oh, S. H. Oh, C. C. Ohm, H. Ohman, H. Oide, H. Okawa, Y. Okumura, T. Okuyama, A. Olariu, L. F. Oleiro Seabra, S. A. Olivares Pino, D. Oliveira Damazio, A. Olszewski, J. Olszowska, A. Onofre, K. Onogi, P. U. E. Onyisi, C. J. Oram, M. J. Oreglia, Y. Oren, D. Orestano, N. Orlando, R. S. Orr, B. Osculati, R. Ospanov, G. Otero y Garzon, H. Otono, M. Ouchrif, F. Ould-Saada, A. Ouraou, K. P. Oussoren, Q. Ouyang, A. Ovcharova, M. Owen, R. E. Owen, V. E. Ozcan, N. Ozturk, K. Pachal, A. Pacheco Pages, C. Padilla Aranda, M. Pagáčová, S. Pagan Griso, F. Paige, P. Pais, K. Pajchel, G. Palacino, S. Palestini, M. Palka, D. Pallin, A. Palma, E. St. Panagiotopoulou, C. E. Pandini, J. G. Panduro Vazquez, P. Pani, S. Panitkin, D. Pantea, L. Paolozzi, Th. D. Papadopoulou, K. Papageorgiou, A. Paramonov, D. Paredes Hernandez, M. A. Parker, K. A. Parker, F. Parodi, J. A. Parsons, U. Parzefall, V. R. Pascuzzi, E. Pasqualucci, S. Passaggio, F. Pastore, Fr. Pastore, G. Pásztor, S. Pataraia, N. D. Patel, J. R. Pater, T. Pauly, J. Pearce, B. Pearson, L. E. Pedersen, M. Pedersen, S. Pedraza Lopez, R. Pedro, S. V. Peleganchuk, D. Pelikan, O. Penc, C. Peng, H. Peng, J. Penwell, B. S. Peralva, M. M. Perego, D. V. Perepelitsa, E. Perez Codina, L. Perini, H. Pernegger, S. Perrella, R. Peschke, V. D. Peshekhonov, K. Peters, R. F. Y. Peters, B. A. Petersen, T. C. Petersen, E. Petit, A. Petridis, C. Petridou, P. Petroff, E. Petrolo, M. Petrov, F. Petrucci, N. E. Pettersson, A. Peyaud, R. Pezoa, P. W. Phillips, G. Piacquadio, E. Pianori, A. Picazio, E. Piccaro, M. Piccinini, M. A. Pickering, R. Piegaia, J. E. Pilcher, A. D. Pilkington, A. W. J. Pin, J. Pina, M. Pinamonti, J. L. Pinfold, A. Pingel, S. Pires, H. Pirumov, M. Pitt, L. Plazak, M.-A. Pleier, V. Pleskot, E. Plotnikova, P. Plucinski, D. Pluth, R. Poettgen, L. Poggioli, D. Pohl, G. Polesello, A. Poley, A. Policicchio, R. Polifka, A. Polini, C. S. Pollard, V. Polychronakos, K. Pommès, L. Pontecorvo, B. G. Pope, G. A. Popeneciu, D. S. Popovic, A. Poppleton, S. Pospisil, K. Potamianos, I. N. Potrap, C. J. Potter, C. T. Potter, G. Poulard, J. Poveda, V. Pozdnyakov, M. E. Pozo Astigarraga, P. Pralavorio, A. Pranko, S. Prell, D. Price, L. E. Price, M. Primavera, S. Prince, M. Proissl, K. Prokofiev, F. Prokoshin, S. Protopopescu, J. Proudfoot, M. Przybycien, D. Puddu, D. Puldon, M. Purohit, P. Puzo, J. Qian, G. Qin, Y. Qin, A. Quadt, W. B. Quayle, M. Queitsch-Maitland, D. Quilty, S. Raddum, V. Radeka, V. Radescu, S. K. Radhakrishnan, P. Radloff, P. Rados, F. Ragusa, G. Rahal, S. Rajagopalan, M. Rammensee, C. Rangel-Smith, M. G. Ratti, F. Rauscher, S. Rave, T. Ravenscroft, M. Raymond, A. L. Read, N. P. Readioff, D. M. Rebuzzi, A. Redelbach, G. Redlinger, R. Reece, K. Reeves, L. Rehnisch, J. Reichert, H. Reisin, C. Rembser, H. Ren, M. Rescigno, S. Resconi, O. L. Rezanova, P. Reznicek, R. Rezvani, R. Richter, S. Richter, E. Richter-Was, O. Ricken, M. Ridel, P. Rieck, C. J. Riegel, J. Rieger, O. Rifki, M. Rijssenbeek, A. Rimoldi, L. Rinaldi, B. Ristić, E. Ritsch, I. Riu, F. Rizatdinova, E. Rizvi, C. Rizzi, S. H. Robertson, A. Robichaud-Veronneau, D. Robinson, J. E. M. Robinson, A. Robson, C. Roda, Y. Rodina, A. Rodriguez Perez, D. Rodriguez Rodriguez, S. Roe, C. S. Rogan, O. Røhne, A. Romaniouk, M. Romano, S. M. Romano Saez, E. Romero Adam, N. Rompotis, M. Ronzani, L. Roos, E. Ros, S. Rosati, K. Rosbach, P. Rose, O. Rosenthal, V. Rossetti, E. Rossi, L. P. Rossi, J. H. N. Rosten, R. Rosten, M. Rotaru, I. Roth, J. Rothberg, D. Rousseau, C. R. Royon, A. Rozanov, Y. Rozen, X. Ruan, F. Rubbo, I. Rubinskiy, V. I. Rud, M. S. Rudolph, F. Rühr, A. Ruiz-Martinez, Z. Rurikova, N. A. Rusakovich, A. Ruschke, H. L. Russell, J. P. Rutherfoord, N. Ruthmann, Y. F. Ryabov, M. Rybar, G. Rybkin, S. Ryu, A. Ryzhov, A. F. Saavedra, G. Sabato, S. Sacerdoti, H. F-W. Sadrozinski, R. Sadykov, F. Safai Tehrani, P. Saha, M. Sahinsoy, M. Saimpert, T. Saito, H. Sakamoto, Y. Sakurai, G. Salamanna, A. Salamon, J. E. Salazar Loyola, D. Salek, P. H. Sales De Bruin, D. Salihagic, A. Salnikov, J. Salt, D. Salvatore, F. Salvatore, A. Salvucci, A. Salzburger, D. Sammel, D. Sampsonidis, A. Sanchez, J. Sánchez, V. Sanchez Martinez, H. Sandaker, R. L. Sandbach, H. G. Sander, M. P. Sanders, M. Sandhoff, C. Sandoval, R. Sandstroem, D. P. C. Sankey, M. Sannino, A. Sansoni, C. Santoni, R. Santonico, H. Santos, I. Santoyo Castillo, K. Sapp, A. Sapronov, J. G. Saraiva, B. Sarrazin, O. Sasaki, Y. Sasaki, K. Sato, G. Sauvage, E. Sauvan, G. Savage, P. Savard, C. Sawyer, L. Sawyer, J. Saxon, C. Sbarra, A. Sbrizzi, T. Scanlon, D. A. Scannicchio, M. Scarcella, V. Scarfone, J. Schaarschmidt, P. Schacht, D. Schaefer, R. Schaefer, J. Schaeffer, S. Schaepe, S. Schaetzel, U. Schäfer, A. C. Schaffer, D. Schaile, R. D. Schamberger, V. Scharf, V. A. Schegelsky, D. Scheirich, M. Schernau, C. Schiavi, C. Schillo, M. Schioppa, S. Schlenker, K. Schmieden, C. Schmitt, S. Schmitt, S. Schmitz, B. Schneider, Y. J. Schnellbach, U. Schnoor, L. Schoeffel, A. Schoening, B. D. Schoenrock, E. Schopf, A. L. S. Schorlemmer, M. Schott, D. Schouten, J. Schovancova, S. Schramm, M. Schreyer, N. Schuh, M. J. Schultens, H.-C. Schultz-Coulon, H. Schulz, M. Schumacher, B. A. Schumm, Ph. Schune, C. Schwanenberger, A. Schwartzman, T. A. Schwarz, Ph. Schwegler, H. Schweiger, Ph. Schwemling, R. Schwienhorst, J. Schwindling, T. Schwindt, G. Sciolla, F. Scuri, F. Scutti, J. Searcy, P. Seema, S. C. Seidel, A. Seiden, F. Seifert, J. M. Seixas, G. Sekhniaidze, K. Sekhon, S. J. Sekula, D. M. Seliverstov, N. Semprini-Cesari, C. Serfon, L. Serin, L. Serkin, M. Sessa, R. Seuster, H. Severini, T. Sfiligoj, F. Sforza, A. Sfyrla, E. Shabalina, N. W. Shaikh, L. Y. Shan, R. Shang, J. T. Shank, M. Shapiro, P. B. Shatalov, K. Shaw, S. M. Shaw, A. Shcherbakova, C. Y. Shehu, P. Sherwood, L. Shi, S. Shimizu, C. O. Shimmin, M. Shimojima, M. Shiyakova, A. Shmeleva, D. Shoaleh Saadi, M. J. Shochet, S. Shojaii, S. Shrestha, E. Shulga, M. A. Shupe, P. Sicho, P. E. Sidebo, O. Sidiropoulou, D. Sidorov, A. Sidoti, F. Siegert, Dj. Sijacki, J. Silva, S. B. Silverstein, V. Simak, O. Simard, Lj. Simic, S. Simion, E. Simioni, B. Simmons, D. Simon, M. Simon, P. Sinervo, N. B. Sinev, M. Sioli, G. Siragusa, S. Yu. Sivoklokov, J. Sjölin, T. B. Sjursen, M. B. Skinner, H. P. Skottowe, P. Skubic, M. Slater, T. Slavicek, M. Slawinska, K. Sliwa, R. Slovak, V. Smakhtin, B. H. Smart, L. Smestad, S. Yu. Smirnov, Y. Smirnov, L. N. Smirnova, O. Smirnova, M. N. K. Smith, R. W. Smith, M. Smizanska, K. Smolek, A. A. Snesarev, G. Snidero, S. Snyder, R. Sobie, F. Socher, A. Soffer, D. A. Soh, G. Sokhrannyi, C. A. Solans Sanchez, M. Solar, E. Yu. Soldatov, U. Soldevila, A. A. Solodkov, A. Soloshenko, O. V. Solovyanov, V. Solovyev, P. Sommer, H. Son, H. Y. Song, A. Sood, A. Sopczak, V. Sopko, V. Sorin, D. Sosa, C. L. Sotiropoulou, R. Soualah, A. M. Soukharev, D. South, B. C. Sowden, S. Spagnolo, M. Spalla, M. Spangenberg, F. Spanò, D. Sperlich, F. Spettel, R. Spighi, G. Spigo, L. A. Spiller, M. Spousta, R. D. St. Denis, A. Stabile, J. Stahlman, R. Stamen, S. Stamm, E. Stanecka, R. W. Stanek, C. Stanescu, M. Stanescu-Bellu, M. M. Stanitzki, S. Stapnes, E. A. Starchenko, G. H. Stark, J. Stark, P. Staroba, P. Starovoitov, S. Stärz, R. Staszewski, P. Steinberg, B. Stelzer, H. J. Stelzer, O. Stelzer-Chilton, H. Stenzel, G. A. Stewart, J. A. Stillings, M. C. Stockton, M. Stoebe, G. Stoicea, P. Stolte, S. Stonjek, A. R. Stradling, A. Straessner, M. E. Stramaglia, J. Strandberg, S. Strandberg, A. Strandlie, M. Strauss, P. Strizenec, R. Ströhmer, D. M. Strom, R. Stroynowski, A. Strubig, S. A. Stucci, B. Stugu, N. A. Styles, D. Su, J. Su, R. Subramaniam, S. Suchek, Y. Sugaya, M. Suk, V. V. Sulin, S. Sultansoy, T. Sumida, S. Sun, X. Sun, J. E. Sundermann, K. Suruliz, G. Susinno, M. R. Sutton, S. Suzuki, M. Svatos, M. Swiatlowski, I. Sykora, T. Sykora, D. Ta, C. Taccini, K. Tackmann, J. Taenzer, A. Taffard, R. Tafirout, N. Taiblum, H. Takai, R. Takashima, H. Takeda, T. Takeshita, Y. Takubo, M. Talby, A. A. Talyshev, J. Y. C. Tam, K. G. Tan, J. Tanaka, R. Tanaka, S. Tanaka, B. B. Tannenwald, S. Tapia Araya, S. Tapprogge, S. Tarem, G. F. Tartarelli, P. Tas, M. Tasevsky, T. Tashiro, E. Tassi, A. Tavares Delgado, Y. Tayalati, A. C. Taylor, G. N. Taylor, P. T. E. Taylor, W. Taylor, F. A. Teischinger, P. Teixeira-Dias, K. K. Temming, D. Temple, H. Ten Kate, P. K. Teng, J. J. Teoh, F. Tepel, S. Terada, K. Terashi, J. Terron, S. Terzo, M. Testa, R. J. Teuscher, T. Theveneaux-Pelzer, J. P. Thomas, J. Thomas-Wilsker, E. N. Thompson, P. D. Thompson, R. J. Thompson, A. S. Thompson, L. A. Thomsen, E. Thomson, M. Thomson, M. J. Tibbetts, R. E. Ticse Torres, V. O. Tikhomirov, Yu. A. Tikhonov, S. Timoshenko, P. Tipton, S. Tisserant, K. Todome, T. Todorov, S. Todorova-Nova, J. Tojo, S. Tokár, K. Tokushuku, E. Tolley, L. Tomlinson, M. Tomoto, L. Tompkins, K. Toms, B. Tong, P. Tornambe, E. Torrence, H. Torres, E. Torró Pastor, J. Toth, F. Touchard, D. R. Tovey, T. Trefzger, L. Tremblet, H. Trepanier, A. Tricoli, I. M. Trigger, S. Trincaz-Duvoid, M. F. Tripiana, W. Trischuk, B. Trocmé, A. Trofymov, C. Troncon, M. Trottier-McDonald, M. Trovatelli, L. Truong, M. Trzebinski, A. Trzupek, J. C-L. Tseng, P. V. Tsiareshka, G. Tsipolitis, N. Tsirintanis, S. Tsiskaridze, V. Tsiskaridze, E. G. Tskhadadze, K. M. Tsui, I. I. Tsukerman, V. Tsulaia, S. Tsuno, D. Tsybychev, A. Tudorache, V. Tudorache, A. N. Tuna, S. A. Tupputi, S. Turchikhin, D. Turecek, D. Turgeman, R. Turra, A. J. Turvey, P. M. Tuts, M. Tylmad, M. Tyndel, G. Ucchielli, I. Ueda, R. Ueno, M. Ughetto, F. Ukegawa, G. Unal, A. Undrus, G. Unel, F. C. Ungaro, Y. Unno, C. Unverdorben, J. Urban, P. Urquijo, P. Urrejola, G. Usai, A. Usanova, L. Vacavant, V. Vacek, B. Vachon, C. Valderanis, E. Valdes Santurio, N. Valencic, S. Valentinetti, A. Valero, L. Valery, S. Valkar, S. Vallecorsa, J. A. Valls Ferrer, W. Van Den Wollenberg, P. C. Van Der Deijl, R. van der Geer, H. van der Graaf, N. van Eldik, P. van Gemmeren, J. Van Nieuwkoop, I. van Vulpen, M. C. van Woerden, M. Vanadia, W. Vandelli, R. Vanguri, A. Vaniachine, P. Vankov, G. Vardanyan, R. Vari, E. W. Varnes, T. Varol, D. Varouchas, A. Vartapetian, K. E. Varvell, F. Vazeille, T. Vazquez Schroeder, J. Veatch, L. M. Veloce, F. Veloso, S. Veneziano, A. Ventura, M. Venturi, N. Venturi, A. Venturini, V. Vercesi, M. Verducci, W. Verkerke, J. C. Vermeulen, A. Vest, M. C. Vetterli, O. Viazlo, I. Vichou, T. Vickey, O. E. Vickey Boeriu, G. H. A. Viehhauser, S. Viel, R. Vigne, M. Villa, M. Villaplana Perez, E. Vilucchi, M. G. Vincter, V. B. Vinogradov, C. Vittori, I. Vivarelli, S. Vlachos, M. Vlasak, M. Vogel, P. Vokac, G. Volpi, M. Volpi, H. von der Schmitt, E. von Toerne, V. Vorobel, K. Vorobev, M. Vos, R. Voss, J. H. Vossebeld, N. Vranjes, M. Vranjes Milosavljevic, V. Vrba, M. Vreeswijk, R. Vuillermet, I. Vukotic, Z. Vykydal, P. Wagner, W. Wagner, H. Wahlberg, S. Wahrmund, J. Wakabayashi, J. Walder, R. Walker, W. Walkowiak, V. Wallangen, C. Wang, C. Wang, F. Wang, H. Wang, H. Wang, J. Wang, J. Wang, K. Wang, R. Wang, S. M. Wang, T. Wang, T. Wang, X. Wang, C. Wanotayaroj, A. Warburton, C. P. Ward, D. R. Wardrope, A. Washbrook, P. M. Watkins, A. T. Watson, I. J. Watson, M. F. Watson, G. Watts, S. Watts, B. M. Waugh, S. Webb, M. S. Weber, S. W. Weber, J. S. Webster, A. R. Weidberg, B. Weinert, J. Weingarten, C. Weiser, H. Weits, P. S. Wells, T. Wenaus, T. Wengler, S. Wenig, N. Wermes, M. Werner, P. Werner, M. Wessels, J. Wetter, K. Whalen, N. L. Whallon, A. M. Wharton, A. White, M. J. White, R. White, S. White, D. Whiteson, F. J. Wickens, W. Wiedenmann, M. Wielers, P. Wienemann, C. Wiglesworth, L. A. M. Wiik-Fuchs, A. Wildauer, H. G. Wilkens, H. H. Williams, S. Williams, C. Willis, S. Willocq, J. A. Wilson, I. Wingerter-Seez, F. Winklmeier, O. J. Winston, B. T. Winter, M. Wittgen, J. Wittkowski, S. J. Wollstadt, M. W. Wolter, H. Wolters, B. K. Wosiek, J. Wotschack, M. J. Woudstra, K. W. Wozniak, M. Wu, M. Wu, S. L. Wu, X. Wu, Y. Wu, T. R. Wyatt, B. M. Wynne, S. Xella, D. Xu, L. Xu, B. Yabsley, S. Yacoob, R. Yakabe, D. Yamaguchi, Y. Yamaguchi, A. Yamamoto, S. Yamamoto, T. Yamanaka, K. Yamauchi, Y. Yamazaki, Z. Yan, H. Yang, H. Yang, Y. Yang, Z. Yang, W-M. Yao, Y. C. Yap, Y. Yasu, E. Yatsenko, K. H. Yau Wong, J. Ye, S. Ye, I. Yeletskikh, A. L. Yen, E. Yildirim, K. Yorita, R. Yoshida, K. Yoshihara, C. Young, C. J. S. Young, S. Youssef, D. R. Yu, J. Yu, J. M. Yu, J. Yu, L. Yuan, S. P. Y. Yuen, I. Yusuff, B. Zabinski, R. Zaidan, A. M. Zaitsev, N. Zakharchuk, J. Zalieckas, A. Zaman, S. Zambito, L. Zanello, D. Zanzi, C. Zeitnitz, M. Zeman, A. Zemla, J. C. Zeng, Q. Zeng, K. Zengel, O. Zenin, T. Ženiš, D. Zerwas, D. Zhang, F. Zhang, G. Zhang, H. Zhang, J. Zhang, L. Zhang, R. Zhang, R. Zhang, X. Zhang, Z. Zhang, X. Zhao, Y. Zhao, Z. Zhao, A. Zhemchugov, J. Zhong, B. Zhou, C. Zhou, L. Zhou, L. Zhou, M. Zhou, N. Zhou, C. G. Zhu, H. Zhu, J. Zhu, Y. Zhu, X. Zhuang, K. Zhukov, A. Zibell, D. Zieminska, N. I. Zimine, C. Zimmermann, S. Zimmermann, Z. Zinonos, M. Zinser, M. Ziolkowski, L. Živković, G. Zobernig, A. Zoccoli, M. zur Nedden, G. Zurzolo, L. Zwalinski

**Affiliations:** 1 Department of Physics, University of Adelaide, Adelaide, Australia; 2 Physics Department, SUNY Albany, Albany, NY USA; 3 Department of Physics, University of Alberta, Edmonton, AB Canada; 4 Department of Physics, Ankara University, Ankara, Turkey; 5 Istanbul Aydin University, Istanbul, Turkey; 6 Division of Physics, TOBB University of Economics and Technology, Ankara, Turkey; 7 LAPP, CNRS/IN2P3 and Université Savoie Mont Blanc, Annecy-le-Vieux, France; 8 High Energy Physics Division, Argonne National Laboratory, Argonne, IL USA; 9 Department of Physics, University of Arizona, Tucson, AZ USA; 10 Department of Physics, The University of Texas at Arlington, Arlington, TX USA; 11 Physics Department, University of Athens, Athens, Greece; 12 Physics Department, National Technical University of Athens, Zografou, Greece; 13 Institute of Physics, Azerbaijan Academy of Sciences, Baku, Azerbaijan; 14 Institut de Física d’Altes Energies (IFAE), The Barcelona Institute of Science and Technology, Barcelona, Spain; 15 Institute of Physics, University of Belgrade, Belgrade, Serbia; 16 Department for Physics and Technology, University of Bergen, Bergen, Norway; 17 Physics Division, Lawrence Berkeley National Laboratory and University of California, Berkeley, CA USA; 18 Department of Physics, Humboldt University, Berlin, Germany; 19 Albert Einstein Center for Fundamental Physics and Laboratory for High Energy Physics, University of Bern, Bern, Switzerland; 20 School of Physics and Astronomy, University of Birmingham, Birmingham, UK; 21 Department of Physics, Bogazici University, Istanbul, Turkey; 22 Department of Physics Engineering, Gaziantep University, Gaziantep, Turkey; 23 Istanbul Bilgi University, Faculty of Engineering and Natural Sciences, Istanbul, Turkey; 24 Bahcesehir University, Faculty of Engineering and Natural Sciences, Istanbul, Turkey; 25 Centro de Investigaciones, Universidad Antonio Narino, Bogota, Colombia; 26 INFN Sezione di Bologna, Bologna, Italy; 27 Dipartimento di Fisica e Astronomia, Università di Bologna, Bologna, Italy; 28 Physikalisches Institut, University of Bonn, Bonn, Germany; 29 Department of Physics, Boston University, Boston, MA USA; 30 Department of Physics, Brandeis University, Waltham, MA USA; 31 Universidade Federal do Rio De Janeiro COPPE/EE/IF, Rio de Janeiro, Brazil; 32 Electrical Circuits Department, Federal University of Juiz de Fora (UFJF), Juiz de Fora, Brazil; 33 Federal University of Sao Joao del Rei (UFSJ), Sao Joao del Rei, Brazil; 34 Instituto de Fisica, Universidade de Sao Paulo, São Paulo, Brazil; 35 Physics Department, Brookhaven National Laboratory, Upton, NY USA; 36 Transilvania University of Brasov, Brasov, Romania; 37 National Institute of Physics and Nuclear Engineering, Bucharest, Romania; 38 Physics Department, National Institute for Research and Development of Isotopic and Molecular Technologies, Cluj Napoca, Romania; 39 University Politehnica Bucharest, Bucharest, Romania; 40 West University in Timisoara, Timisoara, Romania; 41 Departamento de Física, Universidad de Buenos Aires, Buenos Aires, Argentina; 42 Cavendish Laboratory, University of Cambridge, Cambridge, UK; 43 Department of Physics, Carleton University, Ottawa, ON Canada; 44 CERN, Geneva, Switzerland; 45 Enrico Fermi Institute, University of Chicago, Chicago, IL USA; 46 Departamento de Física, Pontificia Universidad Católica de Chile, Santiago, Chile; 47 Departamento de Física, Universidad Técnica Federico Santa María, Valparaiso, Chile; 48 Institute of High Energy Physics, Chinese Academy of Sciences, Beijing, China; 49 Department of Modern Physics, University of Science and Technology of China, Hefei, Anhui China; 50 Department of Physics, Nanjing University, Nanjing, Jiangsu China; 51 School of Physics, Shandong University, Jinan, Shandong China; 52 Shanghai Key Laboratory for Particle Physics and Cosmology, Department of Physics and Astronomy, Shanghai Jiao Tong University (also affiliated with PKU-CHEP), Shanghai, China; 53 Physics Department, Tsinghua University, Beijing, 100084 China; 54 Laboratoire de Physique Corpusculaire, Clermont Université and Université Blaise Pascal and CNRS/IN2P3, Clermont-Ferrand, France; 55 Nevis Laboratory, Columbia University, Irvington, NY USA; 56 Niels Bohr Institute, University of Copenhagen, Copenhagen, Denmark; 57 INFN Gruppo Collegato di Cosenza, Laboratori Nazionali di Frascati, Frascati, Italy; 58 Dipartimento di Fisica, Università della Calabria, Rende, Italy; 59 Faculty of Physics and Applied Computer Science, AGH University of Science and Technology, Kraków, Poland; 60 Marian Smoluchowski Institute of Physics, Jagiellonian University, Kraków, Poland; 61 Institute of Nuclear Physics, Polish Academy of Sciences, Kraków, Poland; 62 Physics Department, Southern Methodist University, Dallas, TX USA; 63 Physics Department, University of Texas at Dallas, Richardson, TX USA; 64 DESY, Hamburg and Zeuthen, Germany; 65 Institut für Experimentelle Physik IV, Technische Universität Dortmund, Dortmund, Germany; 66 Institut für Kern- und Teilchenphysik, Technische Universität Dresden, Dresden, Germany; 67 Department of Physics, Duke University, Durham, NC USA; 68 SUPA-School of Physics and Astronomy, University of Edinburgh, Edinburgh, UK; 69 INFN Laboratori Nazionali di Frascati, Frascati, Italy; 70 Fakultät für Mathematik und Physik, Albert-Ludwigs-Universität, Freiburg, Germany; 71 Section de Physique, Université de Genève, Geneva, Switzerland; 72 INFN Sezione di Genova, Genoa, Italy; 73 Dipartimento di Fisica, Università di Genova, Genoa, Italy; 74 E. Andronikashvili Institute of Physics, Iv. Javakhishvili Tbilisi State University, Tbilisi, Georgia; 75 High Energy Physics Institute, Tbilisi State University, Tbilisi, Georgia; 76 II Physikalisches Institut, Justus-Liebig-Universität Giessen, Giessen, Germany; 77 SUPA-School of Physics and Astronomy, University of Glasgow, Glasgow, UK; 78 II Physikalisches Institut, Georg-August-Universität, Göttingen, Germany; 79 Laboratoire de Physique Subatomique et de Cosmologie, Université Grenoble-Alpes, CNRS/IN2P3, Grenoble, France; 80 Department of Physics, Hampton University, Hampton, VA USA; 81 Laboratory for Particle Physics and Cosmology, Harvard University, Cambridge, MA USA; 82 Kirchhoff-Institut für Physik, Ruprecht-Karls-Universität Heidelberg, Heidelberg, Germany; 83 Physikalisches Institut, Ruprecht-Karls-Universität Heidelberg, Heidelberg, Germany; 84 ZITI Institut für technische Informatik, Ruprecht-Karls-Universität Heidelberg, Mannheim, Germany; 85 Faculty of Applied Information Science, Hiroshima Institute of Technology, Hiroshima, Japan; 86 Department of Physics, The Chinese University of Hong Kong, Shatin, NT Hong Kong; 87 Department of Physics, The University of Hong Kong, Pokfulam, Hong Kong; 88 Department of Physics, The Hong Kong University of Science and Technology, Clear Water Bay, Kowloon, Hong Kong China; 89 Department of Physics, Indiana University, Bloomington, IN USA; 90 Institut für Astro- und Teilchenphysik, Leopold-Franzens-Universität, Innsbruck, Austria; 91 University of Iowa, Iowa City, IA USA; 92 Department of Physics and Astronomy, Iowa State University, Ames, IA USA; 93 Joint Institute for Nuclear Research, JINR Dubna, Dubna, Russia; 94 KEK, High Energy Accelerator Research Organization, Tsukuba, Japan; 95 Graduate School of Science, Kobe University, Kobe, Japan; 96 Faculty of Science, Kyoto University, Kyoto, Japan; 97 Kyoto University of Education, Kyoto, Japan; 98 Department of Physics, Kyushu University, Fukuoka, Japan; 99 Instituto de Física La Plata, Universidad Nacional de La Plata and CONICET, La Plata, Argentina; 100 Physics Department, Lancaster University, Lancaster, UK; 101 INFN Sezione di Lecce, Lecce, Italy; 102 Dipartimento di Matematica e Fisica, Università del Salento, Lecce, Italy; 103 Oliver Lodge Laboratory, University of Liverpool, Liverpool, UK; 104 Department of Physics, Jožef Stefan Institute and University of Ljubljana, Ljubljana, Slovenia; 105 School of Physics and Astronomy, Queen Mary University of London, London, UK; 106 Department of Physics, Royal Holloway University of London, Surrey, UK; 107 Department of Physics and Astronomy, University College London, London, UK; 108 Louisiana Tech University, Ruston, LA USA; 109 Laboratoire de Physique Nucléaire et de Hautes Energies, UPMC and Université Paris-Diderot and CNRS/IN2P3, Paris, France; 110 Fysiska institutionen, Lunds universitet, Lund, Sweden; 111 Departamento de Fisica Teorica C-15, Universidad Autonoma de Madrid, Madrid, Spain; 112 Institut für Physik, Universität Mainz, Mainz, Germany; 113 School of Physics and Astronomy, University of Manchester, Manchester, UK; 114 CPPM, Aix-Marseille Université and CNRS/IN2P3, Marseille, France; 115 Department of Physics, University of Massachusetts, Amherst, MA USA; 116 Department of Physics, McGill University, Montreal, QC Canada; 117 School of Physics, University of Melbourne, Melbourne, VIC Australia; 118 Department of Physics, The University of Michigan, Ann Arbor, MI USA; 119 Department of Physics and Astronomy, Michigan State University, East Lansing, MI USA; 120 INFN Sezione di Milano, Milan, Italy; 121 Dipartimento di Fisica, Università di Milano, Milan, Italy; 122 B.I. Stepanov Institute of Physics, National Academy of Sciences of Belarus, Minsk, Republic of Belarus; 123 National Scientific and Educational Centre for Particle and High Energy Physics, Minsk, Republic of Belarus; 124 Group of Particle Physics, University of Montreal, Montreal, QC Canada; 125 P.N. Lebedev Physical Institute of the Russian, Academy of Sciences, Moscow, Russia; 126 Institute for Theoretical and Experimental Physics (ITEP), Moscow, Russia; 127 National Research Nuclear University MEPhI, Moscow, Russia; 128 D.V. Skobeltsyn Institute of Nuclear Physics, M.V. Lomonosov Moscow State University, Moscow, Russia; 129 Fakultät für Physik, Ludwig-Maximilians-Universität München, Munich, Germany; 130 Max-Planck-Institut für Physik (Werner-Heisenberg-Institut), Munich, Germany; 131 Nagasaki Institute of Applied Science, Nagasaki, Japan; 132 Graduate School of Science and Kobayashi-Maskawa Institute, Nagoya University, Nagoya, Japan; 133 INFN Sezione di Napoli, Naples, Italy; 134 Dipartimento di Fisica, Università di Napoli, Naples, Italy; 135 Department of Physics and Astronomy, University of New Mexico, Albuquerque, NM USA; 136 Institute for Mathematics, Astrophysics and Particle Physics, Radboud University Nijmegen/Nikhef, Nijmegen, The Netherlands; 137 Nikhef National Institute for Subatomic Physics and University of Amsterdam, Amsterdam, The Netherlands; 138 Department of Physics, Northern Illinois University, De Kalb, IL USA; 139 Budker Institute of Nuclear Physics, SB RAS, Novosibirsk, Russia; 140 Department of Physics, New York University, New York, NY USA; 141 Ohio State University, Columbus, OH USA; 142 Faculty of Science, Okayama University, Okayama, Japan; 143 Homer L. Dodge Department of Physics and Astronomy, University of Oklahoma, Norman, OK USA; 144 Department of Physics, Oklahoma State University, Stillwater, OK USA; 145 Palacký University, RCPTM, Olomouc, Czech Republic; 146 Center for High Energy Physics, University of Oregon, Eugene, OR USA; 147 LAL, Univ. Paris-Sud, CNRS/IN2P3, Université Paris Saclay, Orsay, France; 148 Graduate School of Science, Osaka University, Osaka, Japan; 149 Department of Physics, University of Oslo, Oslo, Norway; 150 Department of Physics, Oxford University, Oxford, UK; 151 INFN Sezione di Pavia, Pavia, Italy; 152 Dipartimento di Fisica, Università di Pavia, Pavia, Italy; 153 Department of Physics, University of Pennsylvania, Philadelphia, PA USA; 154 National Research Centre “Kurchatov Institute” B.P.Konstantinov Petersburg Nuclear Physics Institute, St. Petersburg, Russia; 155 INFN Sezione di Pisa, Pisa, Italy; 156 Dipartimento di Fisica E. Fermi, Università di Pisa, Pisa, Italy; 157 Department of Physics and Astronomy, University of Pittsburgh, Pittsburgh, PA USA; 158 Laboratório de Instrumentação e Física Experimental de Partículas-LIP, Lisbon, Portugal; 159 Faculdade de Ciências, Universidade de Lisboa, Lisbon, Portugal; 160 Department of Physics, University of Coimbra, Coimbra, Portugal; 161 Centro de Física Nuclear da Universidade de Lisboa, Lisbon, Portugal; 162 Departamento de Fisica, Universidade do Minho, Braga, Portugal; 163 Departamento de Fisica Teorica y del Cosmos and CAFPE, Universidad de Granada, Granada, Spain; 164 Dep Fisica and CEFITEC of Faculdade de Ciencias e Tecnologia, Universidade Nova de Lisboa, Caparica, Portugal; 165 Institute of Physics, Academy of Sciences of the Czech Republic, Prague, Czech Republic; 166 Czech Technical University in Prague, Prague, Czech Republic; 167 Faculty of Mathematics and Physics, Charles University in Prague, Prague, Czech Republic; 168 State Research Center Institute for High Energy Physics (Protvino), NRC KI, Russia; 169 Particle Physics Department, Rutherford Appleton Laboratory, Didcot, UK; 170 INFN Sezione di Roma, Rome, Italy; 171 Dipartimento di Fisica, Sapienza Università di Roma, Rome, Italy; 172 INFN Sezione di Roma Tor Vergata, Rome, Italy; 173 Dipartimento di Fisica, Università di Roma Tor Vergata, Rome, Italy; 174 INFN Sezione di Roma Tre, Rome, Italy; 175 Dipartimento di Matematica e Fisica, Università Roma Tre, Rome, Italy; 176 Faculté des Sciences Ain Chock, Réseau Universitaire de Physique des Hautes Energies-Université Hassan II, Casablanca, Morocco; 177 Centre National de l’Energie des Sciences Techniques Nucleaires, Rabat, Morocco; 178 Faculté des Sciences Semlalia, Université Cadi Ayyad, LPHEA-Marrakech, Marrakech, Morocco; 179 Faculté des Sciences, Université Mohamed Premier and LPTPM, Oujda, Morocco; 180 Faculté des Sciences, Université Mohammed V, Rabat, Morocco; 181 DSM/IRFU (Institut de Recherches sur les Lois Fondamentales de l’Univers), CEA Saclay (Commissariat à l’Energie Atomique et aux Energies Alternatives), Gif-sur-Yvette, France; 182 Santa Cruz Institute for Particle Physics, University of California Santa Cruz, Santa Cruz, CA USA; 183 Department of Physics, University of Washington, Seattle, WA USA; 184 Department of Physics and Astronomy, University of Sheffield, Sheffield, UK; 185 Department of Physics, Shinshu University, Nagano, Japan; 186 Fachbereich Physik, Universität Siegen, Siegen, Germany; 187 Department of Physics, Simon Fraser University, Burnaby, BC Canada; 188 SLAC National Accelerator Laboratory, Stanford, CA USA; 189 Faculty of Mathematics, Physics and Informatics, Comenius University, Bratislava, Slovak Republic; 190 Department of Subnuclear Physics, Institute of Experimental Physics of the Slovak Academy of Sciences, Kosice, Slovak Republic; 191 Department of Physics, University of Cape Town, Cape Town, South Africa; 192 Department of Physics, University of Johannesburg, Johannesburg, South Africa; 193 School of Physics, University of the Witwatersrand, Johannesburg, South Africa; 194 Department of Physics, Stockholm University, Stockholm, Sweden; 195 The Oskar Klein Centre, Stockholm, Sweden; 196 Physics Department, Royal Institute of Technology, Stockholm, Sweden; 197 Departments of Physics and Astronomy and Chemistry, Stony Brook University, Stony Brook, NY USA; 198 Department of Physics and Astronomy, University of Sussex, Brighton, UK; 199 School of Physics, University of Sydney, Sydney, Australia; 200 Institute of Physics, Academia Sinica, Taipei, Taiwan; 201 Department of Physics, Technion: Israel Institute of Technology, Haifa, Israel; 202 Raymond and Beverly Sackler School of Physics and Astronomy, Tel Aviv University, Tel Aviv, Israel; 203 Department of Physics, Aristotle University of Thessaloniki, Thessaloníki, Greece; 204 International Center for Elementary Particle Physics and Department of Physics, The University of Tokyo, Tokyo, Japan; 205 Graduate School of Science and Technology, Tokyo Metropolitan University, Tokyo, Japan; 206 Department of Physics, Tokyo Institute of Technology, Tokyo, Japan; 207 Department of Physics, University of Toronto, Toronto, ON Canada; 208 TRIUMF, Vancouver, BC Canada; 209 Department of Physics and Astronomy, York University, Toronto, ON Canada; 210 Faculty of Pure and Applied Sciences, and Center for Integrated Research in Fundamental Science and Engineering, University of Tsukuba, Tsukuba, Japan; 211 Department of Physics and Astronomy, Tufts University, Medford, MA USA; 212 Department of Physics and Astronomy, University of California Irvine, Irvine, CA USA; 213 INFN Gruppo Collegato di Udine, Sezione di Trieste, Udine, Italy; 214 ICTP, Trieste, Italy; 215 Dipartimento di Chimica, Fisica e Ambiente, Università di Udine, Udine, Italy; 216 Department of Physics and Astronomy, University of Uppsala, Uppsala, Sweden; 217 Department of Physics, University of Illinois, Urbana, IL USA; 218 Instituto de Física Corpuscular (IFIC) and Departamento de Física Atómica, Molecular y Nuclear and Departamento de Ingeniería Electrónica and Instituto de Microelectrónica de Barcelona (IMB-CNM), University of Valencia and CSIC, Valencia, Spain; 219 Department of Physics, University of British Columbia, Vancouver, BC Canada; 220 Department of Physics and Astronomy, University of Victoria, Victoria, BC Canada; 221 Department of Physics, University of Warwick, Coventry, UK; 222 Waseda University, Tokyo, Japan; 223 Department of Particle Physics, The Weizmann Institute of Science, Rehovot, Israel; 224 Department of Physics, University of Wisconsin, Madison, WI USA; 225 Fakultät für Physik und Astronomie, Julius-Maximilians-Universität, Würzburg, Germany; 226 Fakultät für Mathematik und Naturwissenschaften, Fachgruppe Physik, Bergische Universität Wuppertal, Wuppertal, Germany; 227 Department of Physics, Yale University, New Haven, CT USA; 228 Yerevan Physics Institute, Yerevan, Armenia; 229 Centre de Calcul de l’Institut National de Physique Nucléaire et de Physique des Particules (IN2P3), Villeurbanne, France; 230 CERN, Geneva, Switzerland

## Abstract

A search for strongly produced supersymmetric particles is conducted using signatures involving multiple energetic jets and either two isolated leptons (*e* or $\mu$) with the same electric charge or at least three isolated leptons. The search also utilises *b*-tagged jets, missing transverse momentum and other observables to extend its sensitivity. The analysis uses a data sample of proton–proton collisions at $\sqrt{s}= 13$ TeV recorded with the ATLAS detector at the Large Hadron Collider in 2015 corresponding to a total integrated luminosity of 3.2 fb$^{-1}$. No significant excess over the Standard Model expectation is observed. The results are interpreted in several simplified supersymmetric models and extend the exclusion limits from previous searches. In the context of exclusive production and simplified decay modes, gluino masses are excluded at $95\,\%$ confidence level up to 1.1–1.3 TeV for light neutralinos (depending on the decay channel), and bottom squark masses are also excluded up to 540 GeV. In the former scenarios, neutralino masses are also excluded up to 550–850 GeV for gluino masses around 1 TeV.

## Introduction

Supersymmetry (SUSY) [1–6] is one of the most studied frameworks to extend the Standard Model (SM) beyond the electroweak scale; a general review can be found in Ref. [7]. In its minimal realisation (MSSM) [8, 9] it predicts a new bosonic (fermionic) partner for each fundamental SM fermion (boson), as well as an additional Higgs doublet. If *R*-parity is conserved [10] the lightest supersymmetric particle (LSP) is stable and is typically the lightest neutralino^1^ $\displaystyle \tilde{\chi }^0_1$. Only such scenarios are considered in this paper. In many models, the LSP can be a viable dark matter candidate [11, 12] and produce collider signatures with large missing transverse momentum.

In order to address the SM hierarchy problem with SUSY models [13–16], TeV-scale masses are required [17, 18] for the partners of the gluons (gluinos $\tilde{g}$) and of the top quark chiral degrees of freedom (top squarks $\tilde{t}_{\mathrm {L}}$ and $\tilde{t}_{\mathrm {R}}$), due to the large top Yukawa coupling. The latter also favours significant $\tilde{t}_{\mathrm {L}}$–$\tilde{t}_{\mathrm {R}}$ mixing, so that the lighter mass eigenstate $\tilde{t}_1$ is in many scenarios lighter than the other squarks [19, 20]. Bottom squarks may also be light, being bound to top squarks by $SU(2)_\mathrm{L}$ invariance. This leads to potentially large production cross-sections for gluino pairs ($\tilde{g} \tilde{g}$), top–antitop squark pairs ($\tilde{t}_1 \tilde{t}^{*}_1$) and bottom–antibottom squark pairs ($\tilde{b}_1 \tilde{b}^{*}_1$) at the Large Hadron Collider (LHC) [21]. Production of isolated leptons may arise in the cascade decays of those superpartners to SM quarks and neutralinos $\displaystyle \tilde{\chi }^0_1$, via intermediate neutralinos $\tilde{\chi }^0_{2,3,4}$ or charginos $\tilde{\chi }^\pm _{1,2}$ that in turn lead to *W*, *Z* or Higgs bosons, or to lepton superpartners (sleptons). Lighter third-generation squarks would also enhance $\tilde{g} \rightarrow t\tilde{t}^{*}_1$ or $\tilde{g} \rightarrow b\tilde{b}^{*}_1$ branching ratios over the generic decays involving light-flavour squarks, favouring the production of heavy flavour quarks and, in the case of top quarks, additional leptons.

This paper presents a search for SUSY in final states with two leptons (electrons or muons) of the same electric charge (referred to as same-sign (SS) leptons) [22] or three leptons (3L) in any charge combination, jets and missing transverse momentum (${\vec p}^\mathrm{miss}_\mathrm{T}$, whose magnitude is referred to as $E_{\text {T}}^{\text {miss}}$). It is an extension to $\sqrt{s}=13$ TeV of an earlier search performed by ATLAS with $\sqrt{s}=8$ TeV data [23], and uses the data collected by the ATLAS experiment [24] in proton–proton (*pp*) collisions during 2015. Despite the much lower integrated luminosity collected at $\sqrt{s}=13$ TeV compared to that collected at $\sqrt{s}=8$ TeV, a similar or improved sensitivity at $\sqrt{s}=13$ TeV is expected due to the much larger cross-sections predicted for the production of SUSY particles with masses at the TeV scale. A similar search for SUSY in this topology was also performed by the CMS Collaboration [25] at $\sqrt{s}=8$ TeV. While the same-sign leptons signature is present in many scenarios of physics beyond the SM (BSM), SM processes leading to such final states have very small cross-sections. Compared to many other BSM searches, analyses based on same-sign leptons therefore allow the use of looser kinematic requirements (for example, on $E_{\text {T}}^{\text {miss}}$ or the momentum of jets and leptons), preserving sensitivity to scenarios with small mass differences between gluinos/squarks and the LSP, or in which *R*-parity is not conserved [23].

**Fig. 1:**
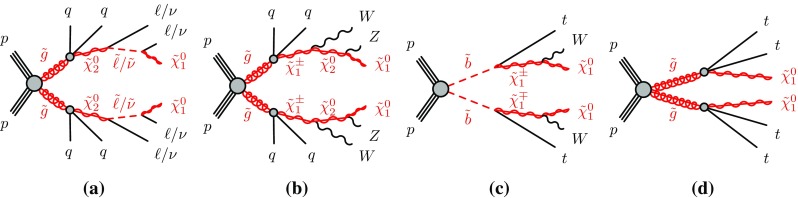
SUSY processes featuring gluino (**a**, **b**, **d**) or bottom squark (**c**) pair production considered in this analysis

The sensitivity to a wide range of models is illustrated by the interpretation of the results in the context of four different SUSY benchmark processes that may lead to same-sign or three-lepton signatures. The first two scenarios focus on gluino pair production with generic decays into light quarks and multiple leptons, either involving light sleptons, $\tilde{g} \rightarrow q\bar{q}\displaystyle \tilde{\chi }^0_2 \rightarrow q\bar{q} \ell \tilde{\ell } ^*\rightarrow q\bar{q}\ell ^+\ell ^-\displaystyle \tilde{\chi }^0_1$ (Fig. 1a), or mediated by a cascade involving $\displaystyle \tilde{\chi }^\pm _1$ and $\displaystyle \tilde{\chi }^0_2$, $\tilde{g} \rightarrow q\bar{q}'\displaystyle \tilde{\chi }^\pm _1 \rightarrow q\bar{q}'W^\pm \displaystyle \tilde{\chi }^0_2 \rightarrow q\bar{q}'W^\pm Z\displaystyle \tilde{\chi }^0_1$ (Fig. 1b). The other two scenarios are motivated by the expectation that the third-generation squarks are lighter than the other squarks and target the direct production of $\tilde{b}_1 \tilde{b}^{*}_1$ pairs with subsequent chargino-mediated $\tilde{b}_1 \rightarrow tW^-\displaystyle \tilde{\chi }^0_1$ decays (Fig. 1c) or the production of $\tilde{g} \tilde{g}$ pairs decaying as $\tilde{g} \rightarrow t\bar{t}\displaystyle \tilde{\chi }^0_1$ via an off-shell top squark (Fig. 1d).

Four signal regions (SRs) are designed to achieve good sensitivity for these SUSY scenarios, mainly characterised by the number of *b*-tagged jets or reconstructed leptons. They are detailed in Sect. 4, preceded by descriptions of the experimental apparatus (Sect. 2) and the simulated samples (Sect. 3). Section 5 is devoted to the estimation of the contribution from SM processes to the signal regions, validated by comparisons with data in dedicated regions. The results are presented in Sect. 6, together with the statistical tests used to interpret the results in the context of the SUSY benchmark scenarios. Finally, Sect. 7 summarises the main conclusions of this paper.

## The ATLAS detector

The ATLAS experiment [24] is a multi-purpose particle detector with a forward-backward symmetric cylindrical geometry and nearly $4\pi$ coverage in solid angle.^2^ The interaction point is surrounded by an inner detector (ID), a calorimeter system, and a muon spectrometer.

The ID provides precision tracking of charged particles for pseudorapidities $|\eta | < 2.5$ and is surrounded by a superconducting solenoid providing a 2 T axial magnetic field. It consists of pixel and silicon-microstrip detectors inside a transition radiation tracker. One significant upgrade for the $\sqrt{s}=13$ TeV running period is the presence of the Insertable B-Layer [26], an additional pixel layer close to the interaction point, which provides high-resolution hits at small radius to improve the tracking performance.

In the pseudorapidity region $|\eta | < 3.2$, high-granularity lead/liquid-argon (LAr) electromagnetic (EM) sampling calorimeters are used. A steel/scintillator tile calorimeter measures hadron energies for $|\eta | < 1.7$. The endcap and forward regions, spanning $1.5<|\eta | <4.9$, are instrumented with LAr calorimeters for both the EM and hadronic measurements.

The muon spectrometer consists of three large superconducting toroids with eight coils each, a system of trigger and precision-tracking chambers, which provide triggering and tracking capabilities in the ranges $|\eta | < 2.4$ and $|\eta | < 2.7$, respectively.

A two-level trigger system is used to select events. The first-level trigger is implemented in hardware and uses a subset of the detector information. This is followed by the software-based High-Level Trigger stage, which can run offline reconstruction and calibration software, reducing the event rate to about 1 kHz.

## Dataset and simulated event samples

The data were collected by the ATLAS detector during 2015 with a peak instantaneous luminosity of $L=5.2\times 10^{33}$ cm$^{-2}$ s$^{-1}$, a bunch spacing of 25 ns, and a mean number of additional *pp* interactions per bunch crossing (pile-up) in the dataset of $\langle \mu \rangle = 14$. After the application of beam, detector and data quality requirements, the integrated luminosity considered in this analysis corresponds to 3.2 fb$^{-1}$ with an uncertainty of $\pm 5\,\%$. The luminosity and its uncertainty are derived following a methodology similar to that detailed in Ref. [27] from a preliminary calibration of the luminosity scale using a pair of *x*–*y* beam separation scans performed in August 2015.

Monte Carlo (MC) simulated event samples are used to aid in the estimation of the background from SM processes and to model the SUSY signal. The MC samples are processed through an ATLAS detector simulation [28] based on Geant4 [29] or a fast simulation using a parameterisation of the calorimeter response and Geant4 for the other parts of the detector [30] and are reconstructed in the same manner as the data.

Diboson processes with four charged leptons ($\ell$), three charged leptons and one neutrino, or two charged leptons and two neutrinos are simulated using the Sherpa  v2.1.1 generator [31], and are described in detail in Ref. [32]. The matrix elements contain the doubly resonant *WW*, *WZ* and *ZZ* processes and all other diagrams with four or six electroweak vertices (such as same-electric-charge *W* boson production in association with two jets, $W^\pm W^\pm jj$). Fully leptonic triboson processes (*WWW*, *WWZ*, *WZZ* and *ZZZ*) with up to six charged leptons are also simulated using Sherpa  v2.1.1 and described in Ref. [32]. The $4\ell$ and $2\ell +2\nu$ processes are calculated at next-to-leading order (NLO) for up to one additional parton; final states with two and three additional partons are calculated at leading order (LO). The $WWZ\rightarrow 4\ell +2\nu$ or $2\ell +4\nu$ processes are calculated at LO with up to two additional partons. The $3\ell +1\nu$ process is calculated at NLO and up to three extra partons at LO using the Comix [33] and OpenLoops [34] matrix element generators and merged with the Sherpa parton shower [35] using the ME+PS@NLO prescription [36]. The $WWW/WZZ\rightarrow 3\ell +3\nu$, $WZZ\rightarrow 5\ell +1\nu$, $ZZZ\rightarrow 6\ell +0\nu$, $4\ell +2\nu$ or $2\ell +4\nu$ processes are calculated with the same configuration but with up to only two extra partons at LO. The CT10 [37] parton distribution function (PDF) set is used for all Sherpa samples in conjunction with a dedicated tuning of the parton shower parameters developed by the Sherpa authors. The generator cross-sections (at NLO for most of the processes) are used when normalising these backgrounds.

Samples of $t\bar{t} V$ (with $V=W$ and *Z*, including non-resonant $Z/\gamma ^*$ contributions) and $t\bar{t}WW$ production are generated at LO with MadGraph v2.2.2 [38] interfaced to the Pythia 8.186 [39] parton shower model, with up to two ($t\bar{t} W$), one ($t\bar{t} Z$) or no ($t\bar{t} WW$) extra partons included in the matrix element; they are described in detail in Ref. [40]. MadGraph is also used to simulate the *tZ*, $t\bar{t} t\bar{t}$ and $t\bar{t} t$ processes. The A14 set of tuned parameters (tune) [41] is used together with the NNPDF23LO PDF set [42]. The $t\bar{t} W$, $t\bar{t} Z$, $t\bar{t} WW$ and $t\bar{t} t\bar{t}$ events are normalised to their NLO cross-section [43] while the generator cross-section is used for *tZ* and $t\bar{t} t$.

Production of a Higgs boson in association with a $t\bar{t}$ pair is simulated using aMC@NLO  [43] (in MadGraph v2.2.2) interfaced to Herwig 2.7.1 [44]. The UEEE5 underlying-event tune is used together with the CTEQ6L1 [45] (matrix element) and CT10 [37] (parton shower) PDF sets. Simulated samples of SM Higgs boson production in association with a *W* or *Z* boson are produced with Pythia 8.186, using the A14 tune and the NNPDF23LO PDF set. Events are normalised with cross-sections calculated at NLO [46].

The signal SUSY processes are generated from LO matrix elements with up to two extra partons, using the MadGraph v2.2.3 generator interfaced to Pythia 8.186 with the A14 tune for the modelling of the SUSY decay chain, parton showering, hadronisation and the description of the underlying event. Parton luminosities are provided by the NNPDF23LO PDF set. Jet–parton matching is realised following the CKKW-L prescription [47], with a matching scale set to one quarter of the pair-produced superpartner mass. Signal cross-sections are calculated to NLO in the strong coupling constant, adding the resummation of soft gluon emission at next-to-leading-logarithmic accuracy (NLO+NLL) [48–52]. The nominal cross-section and the uncertainty are taken from an envelope of cross-section predictions using different PDF sets and factorisation and renormalisation scales, as described in Ref. [53]. The production cross-section of gluino pairs with a mass of 1.2 TeV is 86 fb at $\sqrt{s}=13$ TeV (compared with 4.4 fb at $\sqrt{s}=8$ TeV), while the production cross-section of pairs of bottom squarks with a mass of 500 GeV is 520 fb at $\sqrt{s}=13$ TeV (compared with 86 fb at $\sqrt{s}=8$ TeV).

In all MC samples, except those produced by Sherpa, the EvtGen v1.2.0 program [54] is used to model the properties of the bottom and charm hadron decays. To simulate the effects of additional *pp* collisions in the same and nearby bunch crossings, additional interactions are generated using the soft QCD processes of Pythia 8.186 with the A2 tune [55] and the MSTW2008LO PDF [56], and overlaid onto the simulated hard scatter event. The Monte Carlo samples are reweighted so that the distribution of the number of reconstructed vertices matches the distribution observed in the data.

## Event selection

Candidate events are required to have a reconstructed vertex [57], with at least two associated tracks with $p_{\text {T}} >400$ MeV, and the vertex with the highest sum of squared transverse momentum of the tracks is considered as primary vertex. In order to perform background estimations using data, two categories of electrons and muons are defined: “candidate” and “signal” (the latter being a subset of the “candidate” leptons satisfying tighter selection criteria).

Electron candidates are reconstructed from an isolated electromagnetic calorimeter energy deposit matched to an ID track and are required to have $|\eta |<2.47$, a transverse momentum $p_{\text {T}} >10\,\mathrm {GeV}$, and to pass a loose likelihood-based identification requirement [58, 59]. The likelihood input variables include measurements of calorimeter shower shapes and measurements of track properties from the ID. Candidates within the transition region between the barrel and endcap electromagnetic calorimeters, $1.37<|\eta |<1.52$, are removed. The track matched with the electron must have a significance of the transverse impact parameter with respect to the reconstructed primary vertex, $d_0$, of $\vert d_0\vert /\sigma (d_0) < 5$.

Muon candidates are reconstructed in the region $|\eta |<2.5$ from muon spectrometer tracks matching ID tracks. All muons must have $p_{\text {T}} >10\,\mathrm {GeV}$ and must pass the medium identification requirements defined in Ref. [60], based on selections on the number of hits in the different ID and muon spectrometer subsystems, and the significance of the charge to momentum ratio *q* / *p* [60].

Jets are reconstructed with the anti-$k_t$ algorithm [61] with radius parameter $R=0.4$, using three-dimensional energy clusters in the calorimeter [62] as input. All jets must have $p_{\text {T}} >20\,\mathrm {GeV}$ and $|\eta |<2.8$. Jets are calibrated as described in Ref. [63]. In order to reduce the effects of pile-up, for jets with $p_{\text {T}} <50\,\mathrm {GeV}$ and $|\eta |<2.4$ a significant fraction of the tracks associated with each jet must have an origin compatible with the primary vertex, as defined by the jet vertex tagger [64]. Furthermore, for all jets the expected average energy contribution from pile-up clusters is subtracted according to the jet area [63].

Identification of jets containing *b*-hadrons (*b*-tagging) is performed with the MV2c20 algorithm, a multivariate discriminant making use of track impact parameters and reconstructed secondary vertices [65, 66]. A requirement is chosen corresponding to a 70 % average efficiency obtained for *b*-jets in simulated $t\bar{t}$ events. The rejection factors for light-quark jets, *c*-quark jets and hadronically decaying $\tau$ leptons in simulated $t\bar{t}$ events are approximately 440, 8 and 26, respectively [66]. Jets with $|\eta |<2.5$ which satisfy this *b*-tagging requirement are identified as *b*-jets. To compensate for differences between data and MC simulation in the *b*-tagging efficiencies and mis-tag rates, correction factors are applied to the simulated samples [66].

After object identification, overlaps between objects are resolved. Any jet within a distance $\Delta R_y =\sqrt{(\Delta y)^2+(\Delta \phi )^2}$ = 0.2 of an electron candidate is discarded, unless the jet has a value of the MV2c20 discriminant larger than the value corresponding to approximately an 80 % *b*-tagging efficiency, in which case the electron is discarded since it is likely originating from a semileptonic *b*-hadron decay. Any remaining electron within $\Delta R_y=$ 0.4 of a jet is discarded. Muons within $\Delta R_y=$ 0.4 of a jet are also removed. However, if the jet has fewer than three associated tracks, the muon is kept and the jet is discarded instead to avoid inefficiencies for high-energy muons undergoing significant energy loss in the calorimeter.

Signal electrons must satisfy a tight likelihood-based identification requirement [58, 59] and have $|\eta |<2$ to reduce the impact of electron charge mis-identification. Signal muons must fulfil the requirement of $\vert d_0\vert /\sigma (d_0) < 3$. The track associated to the signal leptons must have a longitudinal impact parameter with respect to the reconstructed primary vertex, $z_0$, satisfying $\vert z_0 \sin \theta \vert < 0.5$ mm. Isolation requirements are applied to both the signal electrons and muons. The scalar sum of the $p_{\text {T}}$ of tracks within a variable-size cone around the lepton, excluding its own track, must be less than 6 % of the lepton $p_{\text {T}}$. The track isolation cone radius for electrons (muons) $\Delta R_\eta =\sqrt{(\Delta \eta )^2+(\Delta \phi )^2}$ is given by the smaller of $\Delta R_\eta = 10\,\mathrm {GeV}/p_{\text {T}}$ and $\Delta R_\eta = 0.2\,(0.3)$, that is, a cone of size $0.2\,(0.3)$ at low $p_{\text {T}}$ but narrower for high-$p_{\text {T}}$ leptons. In addition, in the case of electrons the energy of calorimeter energy clusters in a cone of $\Delta R_\eta = 0.2$ around the electron (excluding the deposition from the electron itself) must be less than 6 % of the electron $p_{\text {T}}$. Simulated events are corrected to account for minor differences in the lepton trigger, reconstruction and identification efficiencies between data and MC simulation.

The missing transverse momentum ${\vec p}^\mathrm{miss}_\mathrm{T}$ is defined as the negative vector sum of the transverse momenta of all identified physics objects (electrons, photons, muons, jets) and an additional soft term. The soft term is constructed from all tracks that are not associated with any physics object, and that are associated with the primary vertex. In this way, the $E_{\text {T}}^{\text {miss}}$ is adjusted for the best calibration of the jets and the other identified physics objects above, while maintaining pile-up independence in the soft term [67, 68].

Events are selected using a combination (logical OR) of dilepton and $E_{\text {T}}^{\text {miss}}$ triggers, the latter being used only for events with $E_{\text {T}}^{\text {miss}} >250\, \mathrm {GeV}$. The trigger-level requirements on $E_{\text {T}}^{\text {miss}}$ and the leading and subleading lepton $p_{\text {T}}$ are looser than those applied offline to ensure that trigger efficiencies are constant in the relevant phase space. Events of interest are selected if they contain at least two signal leptons with $p_{\text {T}} >20$ GeV. If the event contains exactly two signal leptons, they are required to have the same electric charge.

To maximise the sensitivity in different signal models, four overlapping signal regions are defined as shown in Table 1, with requirements on the number of signal leptons ($N_\mathrm{{lept}}^\mathrm{{signal}}$), the number of *b*-jets with $p_{\text {T}} >20\,\mathrm {GeV}$ ($N_{b\mathrm {-jets}}^{20}$), the number of jets with $p_{\text {T}} >50\, \mathrm {GeV}$ regardless of their flavour ($N_\mathrm{{jets}}^{50}$), $E_{\text {T}}^{\text {miss}}$ and the effective mass ($m_{\mathrm {eff}}$), defined as the scalar sum of the $p_{\text {T}}$ of the signal leptons and jets (regardless of their flavour) in the event plus the $E_{\text {T}}^{\text {miss}}$.

**Table 1:** Summary of the event selection criteria for the signal regions (see text for details)

Signal region	$N_\mathrm{{lept}}^\mathrm{{signal}}$	$N_{b{-}\mathrm {jets}}^{20}$	$N_\mathrm{{jets}}^{50}$	$E_{\text {T}}^{\text {miss}}$ (GeV)	$m_{\mathrm {eff}}$ (GeV)
SR0b3j	$\ge$3	$=$0	$\ge$3	$>$200	$>$550
SR0b5j	$\ge$2	$=$0	$\ge$5	$>$125	$>$650
SR1b	$\ge$2	$\ge$1	$\ge$4	$>$150	$>$550
SR3b	$\ge$2	$\ge$3	–	$>$125	$>$650

Each signal region is motivated by a different SUSY scenario. The SR0b3j and SR0b5j signal regions are sensitive to gluino-mediated and directly produced squarks of the first and second generations leading to final states particularly rich in leptons (Fig. 1a) or in jets (Fig. 1b), but with no enhancement of the production of *b*-quarks. Third-generation squark models resulting in final states with two *b*-quarks, such as direct bottom squark production (Fig. 1c), are targeted by the SR1b signal region. Finally, the signal region SR3b targets gluino-mediated top squark production resulting in final states with four *b*-quarks (Fig. 1d).

The values of acceptance times efficiency of the SR selections for the SUSY signal models in Fig. 1 typically range between 1 and 6 % for $m_{\tilde{g}}=1.2\,\mathrm {TeV}$ or $m_{\tilde{b}_1}=600\,\mathrm {GeV}$, and a light $\displaystyle \tilde{\chi }^0_1$.

## Background estimation

Three main sources of SM background can be distinguished in this analysis. A first category consists of events with two same-sign prompt leptons or at least three prompt leptons, mainly from $t\bar{t} V$ and diboson processes. Other types of background events include those containing electrons with mis-measured charge, mainly from the production of top quark pairs, and those containing at least one non-prompt or fake lepton, which mainly originate from hadron decays in events containing top quarks or of *W* bosons in association with jets.

### Background estimation methods

The estimation of the SM background processes with two same-sign prompt leptons or at least three prompt leptons is performed using the MC samples described in Sect. 3. Since diboson and $t\bar{t} V$ events are the main backgrounds in the signal regions, dedicated validation regions with an enhanced contribution from these processes are defined to verify the background predictions (see Sect. 5.3).

Background events due to charge mis-identification, dominated by electrons having emitted a hard bremsstrahlung photon which subsequently converted to an electron–positron pair, are referred to as “charge-flip”. The probability of mis-identifying the charge of a muon is checked in both data and MC simulation, and found to be negligible in the kinematic range relevant to this analysis. The contribution of charge-flip events is estimated using data. The electron charge-flip probability is extracted in a $Z/\gamma ^{*}\rightarrow ee$ data sample using a likelihood fit which takes as input the numbers of same-sign and opposite-sign electron pairs observed in the sample. The charge-flip probability is a free parameter of the fit and is extracted as a function of the electron $p_{\text {T}}$ and $\eta$. The event yield of this background in the signal or validation regions is obtained by applying the measured charge-flip probability to data regions with the same kinematic requirements as the signal or validation regions but with opposite-sign lepton pairs.

The contribution from fake or non-prompt (FNP) leptons (such as hadrons mis-identified as leptons, leptons originating from heavy-flavour decays, and electrons from photon conversions) is also estimated from data with a matrix method similar to that described in Ref. [23]. In this method, two types of lepton identification criteria are defined: “tight”, corresponding to the signal lepton criteria described in Sect. 4, and “loose”, corresponding to candidate leptons. The matrix method relates the number of events containing prompt or FNP leptons to the number of observed events with tight or loose-not-tight leptons using the probability for loose prompt or FNP leptons to satisfy the tight criteria. The probability for loose prompt leptons to satisfy the tight selection criteria is obtained using a $Z/\gamma ^*\rightarrow \ell \ell$ data sample and is modelled as a function of the lepton $p_{\text {T}}$ and $\eta$. The probability for loose FNP leptons to satisfy the tight selection criteria is determined from data in a SS control region enriched in non-prompt leptons originating from heavy-flavour decays. This region contains events with at least one *b*-jet, one tight muon with $p_{\text {T}} > 40\,\mathrm {GeV}$ (likely prompt) and an additional loose electron or muon (likely FNP). The contribution from prompt leptons and charge mis-measured electrons to this region is subtracted from the observed event yields.

The data-driven background estimates are cross-checked with an MC-based technique. In this method, the contributions from processes with FNP leptons and electron charge mis-identification are obtained from MC simulation and normalised to data in dedicated control regions at low jet multiplicity, low $E_{\text {T}}^{\text {miss}}$, and either with or without *b*-jets. The normalisation is performed using five multipliers: one to correct the electron charge mis-identification rate, and four to correct the contributions from FNP electrons or muons originating from *b*-jets or light-flavour jets, respectively. In addition to the MC samples listed in Sect. 3, this method employs samples of top quark pair production generated with the Powheg-Box v2 generator interfaced to Pythia 6.428 [69], as well as samples of simulated *W*+jets and *Z*+jets events generated with Powheg-Box v2 interfaced to Pythia 8.186.

### Systematic uncertainties on the background estimation

Table 2 summarises the contributions of the different sources of systematic uncertainty in the total SM background predictions in the signal regions.

The systematic uncertainties related to the same-sign prompt leptons background estimation arise from the accuracy of the theoretical and experimental modelling in the MC simulation. The primary sources of systematic uncertainties are related to the jet energy scale calibration, the jet energy resolution, *b*-tagging efficiency, and MC modelling and theoretical cross-section uncertainties. The cross-sections used to normalise the MC samples are varied according to the uncertainty in the cross-section calculation, that is, 6 % for diboson, 13 % for $t\bar{t} W$ and 12 % $t\bar{t} Z$ production [43]. Additional uncertainties are assigned to these backgrounds to account for the modelling of the kinematic distributions in the MC simulation. For $t\bar{t} W$ and $t\bar{t} Z$, the predictions from the MadGraph and Sherpa generators are compared, leading to a $\sim$30 % uncertainty for these processes after the SR selections. For dibosons, uncertainties are estimated by varying the renormalisation, factorisation and resummation scales used to generate these samples, leading to a $\sim$30 % uncertainty for these processes after the SR selections. For triboson, $t\bar{t} h$, $t\bar{t} t\bar{t}$ and *tZ* production processes, which constitute a small background in all signal regions, a 50 % uncertainty on the event yields is assumed.

Uncertainties in the FNP lepton background estimate are assigned due to the limited number of data events with loose and tight leptons. In addition, systematic uncertainties of 50–60 % are assigned to the probabilities for loose FNP leptons to satisfy the tight signal criteria to account for potentially different FNP compositions (heavy flavour, light flavour or conversions) between the regions used to measure these probabilities and the SRs, as well as the contamination from prompt leptons in the former regions. This leads to overall FNP background uncertainties in the total background estimates of 18–21 % depending on the signal region.

For the charge-flip background prediction, the main uncertainties originate from the statistical uncertainty of the charge-flip probability measurements and the background contamination of the sample used to extract the charge-flip probability.

**Table 2:** The main sources of systematic uncertainty on the SM background estimates for the four signal regions are shown and their values given as relative uncertainties in the expected signal region background event yields. The individual components can be correlated and therefore do not necessarily add up in quadrature to the total systematic uncertainty. For reference, the total number of expected background events is also shown

	SR0b3j	SR0b5j	SR1b	SR3b
Diboson theoretical uncertainties	23 %	16 %	1 %	$<$1 %
$t\bar{t} V$ theoretical uncertainties	3 %	4 %	13 %	9 %
Other theoretical uncertainties	5 %	3 %	9 %	15 %
MC statistical uncertainties	11 %	14 %	3 %	6 %
Jet energy scale	12 %	11 %	6 %	5 %
Jet energy resolution	3 %	9 %	2 %	3 %
*b*-tagging	4 %	6 %	3 %	10 %
PDF	6 %	6 %	6 %	8 %
Fake/non-prompt leptons	18 %	20 %	18 %	21 %
Charge flip	–	1 %	3 %	8 %
Total background uncertainties	30 %	34 %	22 %	31 %
Total background events	1.5	0.88	4.5	0.80

### Validation of background estimates

**Fig. 2:**
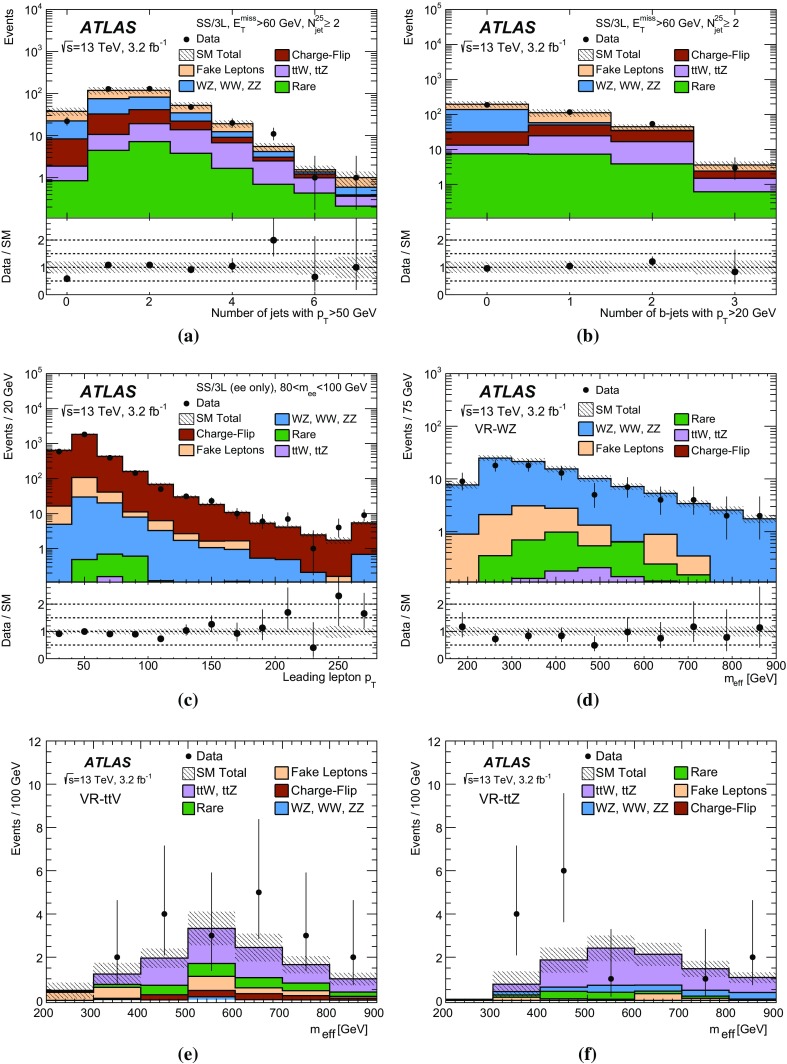
Distributions of kinematic variables after a SS/3L selection including **a**, **b** $E_{\text {T}}^{\text {miss}} >60\,GeV$ and $N_\text {jet}^{25}\ge 2$, **c** a *b*-jet veto and $80<m_{\ell \ell }<100\,\mathrm {GeV}$, and **d**–**f** distributions in the validation regions. The statistical uncertainties in the background prediction are included in the uncertainty band, as well as the theory uncertainties for the backgrounds with prompt SS/3L, and the full systematic uncertainties for data-driven backgrounds. The last bin includes overflows. The “Fake leptons” category corresponds to FNP leptons (see text), and the “Rare” category contains the contributions from associated production of $t\bar{t}$ with $h/WW/t/t\bar{t}$, as well as *tZ*, *Wh*, *Zh*, and triboson production. The *lower part* of the figures **a**–**d** shows the ratio of data to the background prediction

**Fig. 3:**
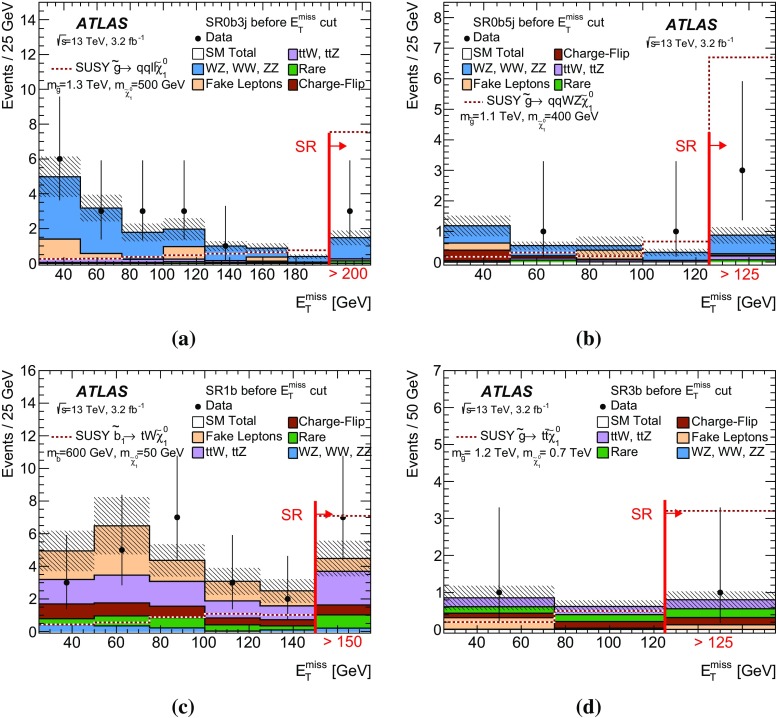
Missing transverse momentum distributions after **a** SR0b3j, **b** SR0b5j, **c** SR1b and **d** SR3b selection, beside the $E_{\text {T}}^{\text {miss}}$ requirement. The results in the signal regions are shown in the last (inclusive) bin of each plot. The statistical uncertainties in the background prediction are included in the uncertainty band, as well as the theory uncertainties for the backgrounds with prompt SS/3L, and the full systematic uncertainties for data-driven backgrounds. The “Fake leptons” category corresponds to FNP leptons (see text), and the “Rare” category contains the contributions from associated production of $t\bar{t}$ with $h/WW/t/t\bar{t}$, as well as *tZ*, *Wh*, *Zh*, and triboson production

To check the validity and robustness of the background estimates, the distributions of several discriminating variables in data are compared with the predicted background after various requirements on the number of jets and *b*-jets. Events are categorised based on the flavours of the selected leptons, and the different flavour channels are compared separately. Examples of such distributions are shown in Fig. 2a, c and illustrate that the predictions and data agree fairly well. The background estimates in a kinematic region close to the signal regions can also be observed in Fig. 3, which shows the $E_{\text {T}}^{\text {miss}}$ distributions in the signal regions before applying the $E_{\text {T}}^{\text {miss}}$ requirements.

**Table 3:** Summary of the event selection in the validation regions. Requirements are placed on the number of signal leptons ($N_\mathrm{{lept}}^\mathrm{{signal}}$) and candidate leptons ($N_\mathrm{{lept}}^\mathrm{{cand}}$), the number of jets with $p_{\text {T}} >25\,\mathrm {GeV}$ ($N_\mathrm{{jets}}^{25}$) or the number of *b*-jets with $p_{\text {T}} >20\,\mathrm{GeV}$ ($N_{b\mathrm {-jets}}^{20}$). The three leading-$p_{\text {T}}$ leptons are referred to as $\ell _{1,2,3}$ with decreasing $p_{\text {T}}$ and the two leading jets as $j_{1,2}$. Additional requirements are set on the invariant mass of the two leading electrons $m_{ee}$, the presence of SS leptons or a pair of same-flavour opposite-sign leptons (SFOS) and its invariant mass $m_\text {SFOS}$

	$N_\mathrm{{lept}}^\mathrm{{signal}}$ ($N_\mathrm{{lept}}^\mathrm{{cand}}$)	$N_{b\mathrm {-jets}}^{20}$	$N_\mathrm{{jets}}^{25}$	$E_{\text {T}}^{\text {miss}}$ (GeV)	$m_{\mathrm {eff}}$ (GeV)	Other
VR-WW	=2 (=2)	=0	$\ge$2	35–200	300–900	$m(j_1 j_2)>500$ GeV
	=1 SS pair					$p_{\text {T}} (j_2)>40$ GeV
						$p_{\text {T}} (\ell _2)>30$ GeV
						veto $80<m_{ee}<100$ GeV
VR-WZ	=3 (=3)	=0	1–3	30–200	$<$900	$p_{\text {T}} (\ell _3)>30$ GeV
VR-ttV	$\ge$2 ($-$)	$\ge$2	$\ge$5 ($e^\pm e^\pm$,$e^\pm \mu ^\pm$)	20–200	200–900	$p_{\text {T}} (\ell _2)>25$ GeV
	$\ge$1 SS pair		$\ge$3 ($\mu ^\pm \mu ^\pm$)			veto $\{E_{\text {T}}^{\text {miss}} >125$ and $m_{\mathrm {eff}}>650\,\mathrm {GeV}\}$
VR-ttZ	$\ge$3 ($-$)	$\ge$1	$\ge$4 ($=$1 *b*-jet)	20–150	100–900	$p_{\text {T}} (\ell _2)>25$ GeV
	$\ge$1 SFOS pair		$\ge$3 ($\ge$2 *b*-jets)			$p_{\text {T}} (\ell _3)>20$ GeV (if *e*)
						$80<m_\text {SFOS}<100$ GeV
All VRs	Veto events belonging to any SR, or if $\ell _1$ or $\ell _2$ is an electron with $|\eta |>1.37$ (except in VR-WZ)					

Dedicated validation regions (VRs) are defined to test the estimate of the rare SM processes contributing to the signal regions, whose cross-sections have not yet been measured at $\sqrt{s}=13$ TeV. The corresponding selections are summarised in Table 3. In these regions, upper bounds are placed on $E_{\text {T}}^{\text {miss}}$ and $m_{\mathrm {eff}}$ to reduce signal contamination, and the small residual overlap with the signal regions is resolved by vetoing events that contribute to the signal regions. To further reduce contributions from electron charge mis-identification, events are also vetoed if one of the two leading leptons is an electron with $|\eta |>1.37$, since contributions from charge-flip electrons are smaller in the central region due to the lower amount of crossed material. The purity of the targeted processes in these regions ranges from about 40 to $80\,\%$. The VR-ttV and VR-ttZ regions overlap with each other, with 30 % of the $t\bar{t} V$ events in VR-ttV also present in VR-ttZ, and the contributions from the $t\bar{t} Z$ and $t\bar{t} W$ processes is similar in VR-ttV.

The observed yields in these validation regions, compared with the background predictions and uncertainties, can be seen in Table 4, and the effective mass distributions are shown in Fig. 2d, f. There is fair agreement between data and the estimated background for the validation regions, with the largest deviations being observed in VR-ttV with a 1.5$\sigma$ deviation.

**Table 4:** The numbers of observed data and expected background events for the validation regions. The “Rare” category contains the contributions from associated production of $t\bar{t}$ with $h/WW/t/t\bar{t}$, as well as *tZ*, *Wh*, *Zh*, and triboson production. Background categories shown as “$-$” denote that they cannot contribute to a given region (charge flips or $W^\pm W^\pm jj$ in 3-lepton regions). The individual uncertainties can be correlated and therefore do not necessarily add up in quadrature to the total systematic uncertainty

	VR-WW	VR-WZ	VR-ttV	VR-ttZ
Observed events	4	82	19	14
Total background events	$3.4 \pm 0.8$	$98 \pm 15$	$12.1 \pm 2.7$	$9.7 \pm 2.5$
Fake/non-prompt leptons	$0.6 \pm 0.5$	$8 \pm 6$	$2.1 \pm 1.4$	$0.6\pm 1.0$
Charge-flip	$0.26 \pm 0.05$	$-$	$1.14 \pm 0.15$	$-$
$t\bar{t}W$	$0.05 \pm 0.03$	$0.25 \pm 0.09$	$2.4 \pm 0.8$	$0.10 \pm 0.03$
$t\bar{t}Z$	$0.02 \pm 0.01$	$0.72 \pm 0.26$	$3.9 \pm 1.3$	$6.3 \pm 2.1$
*WZ*	$1.0 \pm 0.4$	$78 \pm 13$	$0.19 \pm 0.10$	$1.2 \pm 0.4$
$W^\pm W^\pm jj$	$1.3 \pm 0.5$	$-$	$0.02 \pm 0.03$	$-$
*ZZ*	$0.02 \pm 0.01$	$8.2 \pm 2.8$	$0.12 \pm 0.15$	$0.30 \pm 0.19$
Rare	$0.10 \pm 0.05$	$2.8 \pm 1.4$	$2.3 \pm 1.2$	$1.1 \pm 0.6$

## Results

**Table 5:** The number of observed data events and expected background contributions in the signal regions. The *p*-value of the observed events for the background-only hypothesis is denoted by $p(s = 0)$. The “Rare” category contains the contributions from associated production of $t\bar{t}$ with $h/WW/t/t\bar{t}$, as well as *tZ*, *Wh*, *Zh*, and triboson production. Background categories shown as “$-$” denote that they cannot contribute to a given region (charge flips or $W^\pm W^\pm jj$ in 3-lepton regions). The individual uncertainties can be correlated and therefore do not necessarily add up in quadrature to the total systematic uncertainty

	SR0b3j	SR0b5j	SR1b	SR3b
Observed events	3	3	7	1
Total background events	$1.5 \pm 0.4$	$0.88 \pm 0.29$	$4.5 \pm 1.0$	$0.80 \pm 0.25$
$p(s = 0)$	0.13	0.04	0.15	0.36
Fake/non-prompt leptons	$<$0.2	$0.05\pm 0.18$	$0.8 \pm 0.8$	$0.13 \pm 0.17$
Charge-flip	$-$	$0.02 \pm 0.01$	$0.60 \pm 0.12$	$0.19 \pm 0.06$
$t\bar{t}W$	$0.02 \pm 0.01$	$0.08 \pm 0.04$	$1.1 \pm 0.4$	$0.10 \pm 0.05$
$t\bar{t}Z$	$0.10 \pm 0.04$	$0.05 \pm 0.03$	$0.92 \pm 0.31$	$0.14 \pm 0.06$
*WZ*	$1.2 \pm 0.4$	$0.48 \pm 0.20$	$0.18 \pm 0.11$	$<$0.02
$W^\pm W^\pm jj$	$-$	$0.12 \pm 0.07$	$0.03 \pm 0.02$	$<$0.01
*ZZ*	$<$0.03	$<$0.04	$<$0.03	$<$0.03
Rare	$0.14 \pm 0.08$	$0.07 \pm 0.05$	$0.8 \pm 0.4$	$0.24 \pm 0.14$

Figure 3 shows the data $E_{\text {T}}^{\text {miss}}$ distributions after the signal region selections (beside that on $E_{\text {T}}^{\text {miss}}$) in data together with the expected contributions from all the SM backgrounds with their total statistical and systematic uncertainties. For illustration, a typical SUSY signal distribution corresponding to the most relevant benchmark scenario in each SR is displayed. The detailed yields for data and the different sources of SM background in the signal regions are presented in Table 5. The uncertainties amount to 22–34 % of the total background depending on the signal region. In all four SRs the number of data events exceeds the expectation but is consistent within the uncertainties, the smallest *p*-value for the SM-only hypothesis being 0.04 for SR0b5j. Out of the 14 events in the SRs, 2 of the events in SR1b and the 3 events in SR0b3j contain three leptons. None of those events contain three leptons of equal charge, or are present in more than one SR.

In the absence of any significant deviations from the SM predictions, upper limits on possible BSM contributions to the signal regions are computed, in particular in the context of the SUSY benchmark scenarios described in Sect. 1. The HistFitter framework [70], which utilises a profile-likelihood-ratio test [71], is used to establish 95 % confidence intervals using the CL$_\mathrm {s}$ prescription [72]. The likelihood is built as the product of a Poisson probability density function describing the observed number of events in the signal region and Gaussian distributions constraining the nuisance parameters associated with the systematic uncertainties whose widths correspond to the sizes of these uncertainties; Poisson distributions are used instead for MC statistical uncertainties. Correlations of a given nuisance parameter across the different sources of backgrounds and the signal are taken into account when relevant. The statistical tests are performed independently for each of the signal regions.

Table 6 presents 95 % confidence level (CL) model-independent upper limits on the number of BSM events, $N_\mathrm {BSM}$, that may contribute to the signal regions. Normalising these by the integrated luminosity *L* of the data sample, they can be interpreted as upper limits on the visible BSM cross-section $\sigma _\mathrm{{vis}}$, defined as the product $\sigma _\mathrm{{prod}}\times A \times \epsilon =N_\mathrm {BSM}/L$ of production cross-section, acceptance and reconstruction efficiency.

**Table 6:** Signal model-independent upper limits on the number of BSM events ($N_\mathrm{{BSM}}$) and the visible signal cross-section ($\sigma _\mathrm{{vis}}$) in the four SRs. The numbers (in parentheses) give the observed (expected under the SM hypothesis) 95 % CL upper limits. Calculations are performed with pseudo-experiments. The $\pm$1$\sigma$ variations on the expected limit due to the statistical and systematic uncertainties in the background prediction are also shown

	SR0b3j	SR0b5j	SR1b	SR3b
$N_\mathrm{{BSM}}^\mathrm{{obs}}$ ($N_\mathrm{{BSM}}^\mathrm{{exp}}$)	5.9 $({4.1}^{+1.6}_{-0.8})$	6.4 $({3.6}^{+1.2}_{-1.1})$	8.8 $({6.0}^{+2.6}_{-1.6})$	3.8 $({3.7}^{+1.1}_{-0.5})$
$\sigma _\mathrm{{vis}}^\mathrm{{obs}}$ [fb]	1.8	2.0	2.8	1.2

**Fig. 4:**
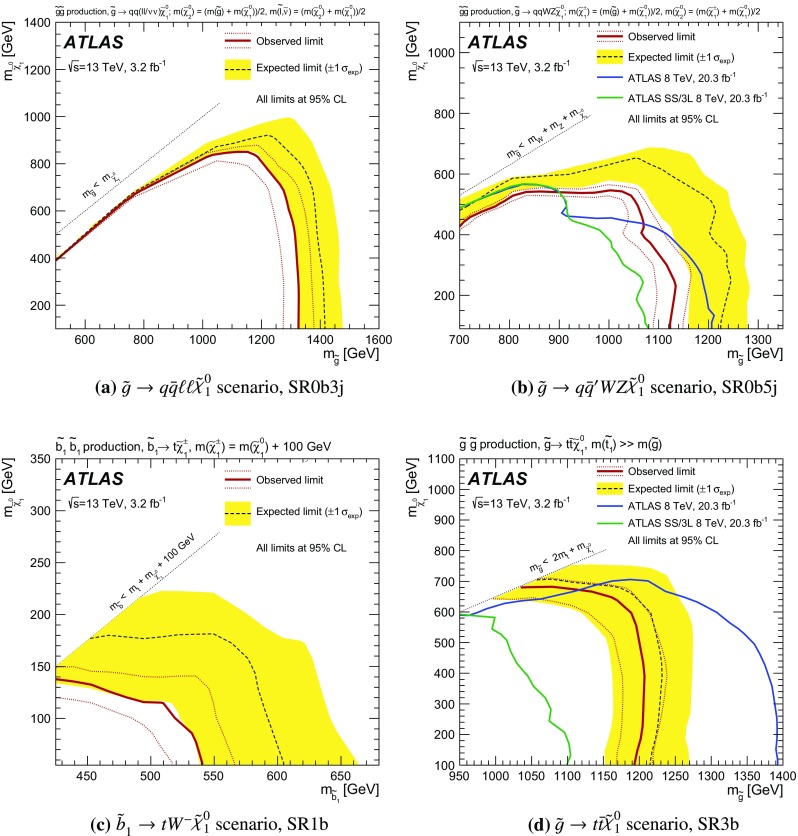
Observed and expected exclusion limits on the $\tilde{g}$, $\tilde{b}_1$ and $\displaystyle \tilde{\chi }^0_1$ masses in the context of SUSY scenarios with simplified mass spectra featuring $\tilde{g} \tilde{g}$ or $\tilde{b}_1 \tilde{b}^{*}_1$ pair production with exclusive decay modes. The signal region used to obtain the limits is specified for each scenario. The contours of the band around the expected limit are the $\pm$1$\sigma$ results, including all uncertainties except theoretical uncertainties on the signal cross-section. The *dotted lines* around the observed limit illustrate the change in the observed limit as the nominal signal cross-section is scaled up and down by the theoretical uncertainty. All limits are computed at 95 % CL. The *diagonal lines* indicate the kinematic limit for the decays in each specified scenario. For figures **b**, **d** Results are compared with the observed limits obtained by previous ATLAS searches [23, 73, 74]. For figures **a**, **c** a direct comparison with earlier searches is not possible, due to differing model assumptions

Exclusion limits are also set on the masses of the superpartners involved in the four SUSY benchmark scenarios considered in this analysis. Simplified models corresponding to a single production mode and with 100 % branching ratio to a specific decay chain are used, with the masses of the SUSY particles not involved in the process set to very high values. Figure 4 shows the limits on the mass of the $\displaystyle \tilde{\chi }^0_1$ as a function of the $\tilde{g}$ or $\tilde{b}_1$ mass. In some cases, the new limits set by this analysis can be compared with the existing limits set by the combination of ATLAS SUSY searches with 8 TeV data [73, 74]. For parts of the parameter space, the sensitivity reached with the 13 TeV dataset exceeds that of the 8 TeV dataset, and additional parameter space regions can be excluded, especially for large neutralino masses.

Signal models featuring gluino pair production with a subsequent gluino decay via $\displaystyle \tilde{\chi }^0_2$ and light sleptons ($\tilde{g} \rightarrow q\bar{q}\displaystyle \tilde{\chi }^0_2 \rightarrow q\bar{q} (\ell \tilde{\ell } ^*/\nu \tilde{\nu }^*)\rightarrow q\bar{q}(\ell ^+\ell ^-/\nu \nu )\displaystyle \tilde{\chi }^0_1$) are probed using SR0b3j (Fig. 4a). In this simplified model, the gluinos decay into $u\bar{u}$, $d\bar{d}$, $s\bar{s}$ or $c\bar{c}$ with equal probabilities, and the six types of leptons are also produced in the $\tilde{\chi }_2^0$ decays with equal probabilities. The $\displaystyle \tilde{\chi }^0_2$ mass is set to $m_{\displaystyle \tilde{\chi }^0_2}=(m_{\tilde{g}} + m_{\displaystyle \tilde{\chi }^0_1})/2$, with the $\tilde{\ell }$ and $\tilde{\nu }$ masses set to $m_{\tilde{\ell },\tilde{\nu }}=(m_{\displaystyle \tilde{\chi }^0_2} + m_{\displaystyle \tilde{\chi }^0_1})/2$. Gluino masses up to $m_{\tilde{g}}\approx 1.3\,\mathrm {TeV}$ for a light $\displaystyle \tilde{\chi }^0_1$ and $\displaystyle \tilde{\chi }^0_1$ masses up to $m_{\displaystyle \tilde{\chi }^0_1}\approx 850\,\mathrm {GeV}$ for gluinos with $m_{\tilde{g}}\approx 1.1\, \mathrm {TeV}$ are excluded in this scenario.

Similarly, models with gluino production with a subsequent two-step gluino decay via $\displaystyle \tilde{\chi }^\pm _1$ and $\displaystyle \tilde{\chi }^0_2$ ($\tilde{g} \rightarrow q\bar{q} \displaystyle \tilde{\chi }^\pm _1 \rightarrow q\bar{q} W\displaystyle \tilde{\chi }^0_2 \rightarrow q\bar{q} W Z \displaystyle \tilde{\chi }^0_1$) are probed with SR0b5j (Fig. 4b). In this simplified model, the gluinos decay into $u\bar{u}$, $d\bar{d}$, $s\bar{s}$ or $c\bar{c}$ with equal probabilities. The $\displaystyle \tilde{\chi }^\pm _1$ mass is set to $m_{\displaystyle \tilde{\chi }^\pm _1}=(m_{\tilde{g}} + m_{\displaystyle \tilde{\chi }^0_1})/2$ and the $\displaystyle \tilde{\chi }^0_2$ mass is set to $m_{\displaystyle \tilde{\chi }^0_2}=(m_{\displaystyle \tilde{\chi }^\pm _1} + m_{\displaystyle \tilde{\chi }^0_1})/2$; *W* and *Z* bosons produced in the decay chain are not necessarily on-shell. The exclusion limits in this scenario reach $m_{\tilde{g}}\approx 1.1\,\mathrm {TeV}$ (for light $\displaystyle \tilde{\chi }^0_1$) and $m_{\displaystyle \tilde{\chi }^0_1}\approx 550\,\mathrm {GeV}$ (for $m_{\tilde{g}}\approx 1.0\,\mathrm {TeV}$).

Exclusion limits in a simplified model of bottom squark production with chargino-mediated $\tilde{b}_1 \rightarrow tW^-\displaystyle \tilde{\chi }^0_1$ decays are obtained with SR1b (Fig. 4c). In this model the $\displaystyle \tilde{\chi }^\pm _1$ mass is set to $m_{\displaystyle \tilde{\chi }^\pm _1}=m_{\displaystyle \tilde{\chi }^0_1} + 100\,\mathrm {GeV}$. The limits can reach mass values of $m_{\tilde{b}_1}\approx 540\,\mathrm {GeV}$ for a light $\displaystyle \tilde{\chi }^0_1$, while $m_{\displaystyle \tilde{\chi }^0_1}\lesssim 140\,\mathrm {GeV}$ are also excluded for $m_{\tilde{b}_1}\approx 425\,\mathrm {GeV}$, significantly extending the previous limits obtained at $\sqrt{s}=8$ TeV [74] which excluded $m_{\tilde{b}_1}\lesssim 470\,\mathrm {GeV}$ for $m_{\displaystyle \tilde{\chi }^0_1}\approx 60\,\mathrm {GeV}$ for a similar model.

Finally, SR3b is used to set limits on masses in a simplified model with gluino pair production and $\tilde{g} \rightarrow t\bar{t}\displaystyle \tilde{\chi }^0_1$ decays via an off-shell top squark (Fig. 4d). In that case, gluino masses of $m_{\tilde{g}}\lesssim 1.2\,\mathrm {TeV}$ are excluded for $m_{\displaystyle \tilde{\chi }^0_1}\lesssim 600\,\mathrm {GeV}$, and $\displaystyle \tilde{\chi }^0_1$ masses up to $m_{\displaystyle \tilde{\chi }^0_1}\approx 680\,\mathrm {GeV}$ are also excluded for $m_{\tilde{g}}\approx 1.05\,\mathrm {TeV}$.

## Conclusion

A search for supersymmetry in events with exactly two same-sign leptons or at least three leptons, multiple jets, *b*-jets and $E_{\text {T}}^{\text {miss}}$ is presented. The analysis is performed with proton–proton collision data at $\sqrt{s}=13\mathrm {TeV}$ collected with the ATLAS detector at the Large Hadron Collider corresponding to an integrated luminosity of 3.2 fb$^{-1}$. With no significant excess over the Standard Model expectation observed, results are interpreted in the framework of simplified models featuring gluino and bottom squark production. In the $\tilde{g} \tilde{g}$ simplified models considered, $m_{\tilde{g}}\lesssim 1.1$–$1.3~\mathrm {TeV}$ and $m_{\displaystyle \tilde{\chi }^0_1}\lesssim 550$–$850~\mathrm {GeV}$ are excluded at 95 % confidence level depending on the model parameters. Bottom squark masses of $m_{\tilde{b}_1}\lesssim 540~\mathrm {GeV}$ are also excluded for a light $\displaystyle \tilde{\chi }^0_1$ in a $\tilde{b}_1 \tilde{b}^{*}_1$ simplified model with $\tilde{b}_1 \rightarrow tW^-\displaystyle \tilde{\chi }^0_1$. These results are complementary to those of previous searches and extend the exclusion limits they set.

## Footnotes

1 The SUSY partners of the Higgs and electroweak gauge bosons mix to form the mass eigenstates known as charginos ($\tilde{\chi }^{\pm }_{l}$, $l = 1, 2$ ordered by increasing mass) and neutralinos ($\tilde{\chi }^{0}_{m}$, $m = 1, \ldots , 4$ ordered by increasing mass).

2 ATLAS uses a right-handed coordinate system with its origin at the nominal interaction point (IP) in the centre of the detector and the *z*-axis along the beam pipe. The *x*-axis points from the IP to the centre of the LHC ring, and the *y*-axis points upward. Cylindrical coordinates (*r*, $\phi$) are used in the transverse plane, $\phi$ being the azimuthal angle around the beam pipe. The pseudorapidity is defined in terms of the polar angle $\theta$ as $\eta = -\ln \tan (\theta /2)$. Rapidity is defined as $y=0.5 \ln \left[ (E + p_z )/(E - p_z )\right]$ where *E* denotes the energy and $p_z$ is the component of the momentum along the beam direction.
